# Design, Synthesis and in Combo Antidiabetic Bioevaluation of Multitarget Phenylpropanoic Acids †

**DOI:** 10.3390/molecules23020340

**Published:** 2018-02-06

**Authors:** Blanca Colín-Lozano, Samuel Estrada-Soto, Fabiola Chávez-Silva, Abraham Gutiérrez-Hernández, Litzia Cerón-Romero, Abraham Giacoman-Martínez, Julio Cesar Almanza-Pérez, Emanuel Hernández-Núñez, Zhilong Wang, Xin Xie, Mario Cappiello, Francesco Balestri, Umberto Mura, Gabriel Navarrete-Vazquez

**Affiliations:** 1 Facultad de Farmacia, Universidad Autónoma del Estado de Morelos, Cuernavaca, Morelos 62209, Mexico; clbi_ff@uaem.mx (B.C.-L.); enoch@uaem.mx (S.E.-S.); facasy@gmail.com (F.C.-S.); ghaa_ff@uaem.mx (A.G.-H.); crlc_ff@uaem.mx (L.C.-R.); 2 Laboratorio de Farmacología, Departamento de Ciencias de la Salud, Universidad Autónoma Metropolitana Iztapalapa, Ciudad de México 09340, Mexico; agmfest@hotmail.com (A.G.-M.); j.almanza.perez@gmail.com (J.C.A.-P.); 3 Cátedra CONACyT, Departamento de Recursos del Mar, Centro de Investigación y de Estudios Avanzados del IPN, Unidad Mérida, Yucatán 97310, Mexico; emanuel.hernandez@cinvestav.mx; 4 CAS Key Laboratory of Receptor Research, the National Center for Drug Screening, Shanghai Institute of Materia Medica, Chinese Academy of Sciences, Shanghai 201203, China, endlesslily@hotmail.com (Z.W.); xxie@simm.ac.cn (X.X.); 5 Dipartimento di Biologia, Unità di Biochimica, University of Pisa, 56126 Pisa, Italy; mcappiello@biologia.unipi.it (M.C.); francesco.balestri@unipi.it (F.B.); umberto.mura@unipi.it (U.M.)

**Keywords:** diabetes, GPR40, AKRB1, PPARγ, GLUT-4

## Abstract

We have synthesized a small series of five 3-[4-arylmethoxy)phenyl]propanoic acids employing an easy and short synthetic pathway. The compounds were tested in vitro against a set of four protein targets identified as key elements in diabetes: G protein-coupled receptor 40 (GPR40), aldose reductase (AKR1B1), peroxisome proliferator-activated receptor gama (PPARγ) and solute carrier family 2 (facilitated glucose transporter), member 4 (GLUT-4). Compound **1** displayed an EC_50_ value of 0.075 μM against GPR40 and was an AKR1B1 inhibitor, showing IC_50_ = 7.4 μM. Compounds **2** and **3** act as slightly AKR1B1 inhibitors, potent GPR40 agonists and showed an increase of 2 to 4-times in the mRNA expression of PPARγ, as well as the GLUT-4 levels. Docking studies were conducted in order to explain the polypharmacological mode of action and the interaction binding mode of the most active molecules on these targets, showing several coincidences with co-crystal ligands. Compounds **1**–**3** were tested in vivo at an explorative 100 mg/kg dose, being **2** and **3** orally actives, reducing glucose levels in a non-insulin-dependent diabetes mice model. Compounds **2** and **3** displayed robust in vitro potency and in vivo efficacy, and could be considered as promising multitarget antidiabetic candidates. This is the first report of a single molecule with these four polypharmacological target action.

## 1. Introduction

**Figure 1:**
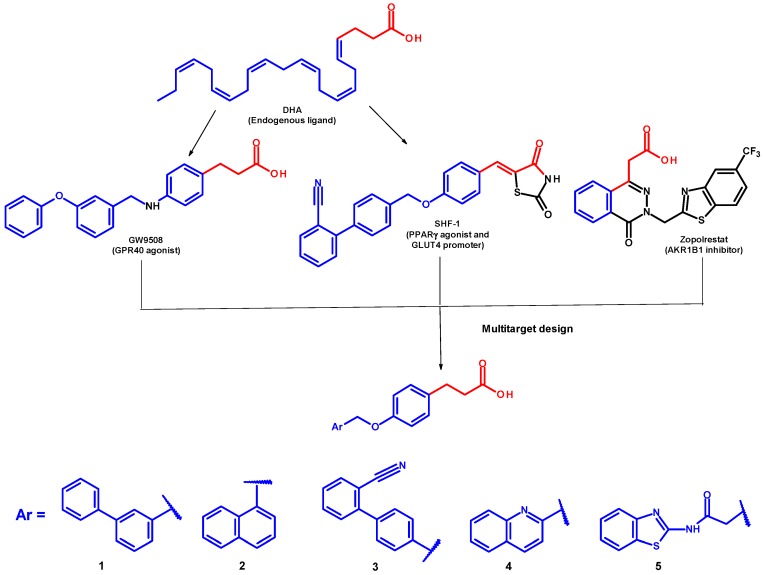
Drug design of multitarget compounds **1**–**5**, which were designed from pharmacophore extraction and reorganization from known modulators of four targets: GPR40, PPARγ, GLUT-4, and AKR1B1.

Type 2 Diabetes Mellitus (T2DM) is a multifactorial metabolic disease that occurs with fasting blood glucose concentrations above 120 mg/dL (>7 mM), due to the abnormal pancreatic β-cells and/or insulin resistance [1]. Currently, experimental drug discovery for T2DM is focused on developing drugs for insulin sensitizing and/or releasing effect by several mechanisms. One of them is mediated by the G protein-coupled receptor 40 (GPR40 or FFA1), which is primarily expressed in pancreatic β-cells and enteroendocrine cells of the gut [2,3]. The GPR40 activation by medium to long chain fatty acids, stimulates insulin secretion only in the presence of high glucose concentration but does not disturb insulin exocytosis at low glucose levels [4,5]. This intriguing mechanism was suggestive in treating T2DM by small molecules agonists for GPR40, thus playing as novel insulin secretagogues with little or no risk of hypoglycemia [6]. An alternative mechanism for the control of glucose levels refers to the peroxisome proliferator-activated receptors (PPARs) action. PPARs, members of the nuclear hormone receptor family, occur in three subtypes: PPARα, PPARγ, and PPARβ/δ [7]. Each subtype regulates tissue-specific target genes acting as lipid sensors and regulators of glucose homeostasis, such as the solute carrier family 2 (facilitated glucose transporter), member 4 (GLUT-4) [8]. Glitazones function as insulin-sensitizing drugs, via activation of PPARγ [9,10], leading to enforced expression of target genes involved in glucose-sensing ability of pancreatic β-cells in diabetic subjects [11]. On the other hand, extended hyperglycemia plays a significant key in the development of diabetic complications: atherosclerosis, blindness, neuropathy and renal failure. The NADPH-dependent reduction of elevated levels of d-glucose to sorbitol catalyzed by aldose reductase (AKR1B1) is considered as one of the phenomena leading to the onset of long term diabetic complications [12]. Stressful and damaging cell conditions are produced by several factors such as the osmotic imbalance due to sorbitol accumulation, the loss of antioxidant power related with NADPH oxidation and the induction of additional advantageous conditions for protein glycation [13,14,15]. Inhibition of AKR1B1 represents a potential target of antidiabetic drug action. The purpose of the present work was to design, synthesize and screen in vitro, in vivo and in silico (in combo screens) a small series of five 3-[4-arylmethoxy)phenyl]propanoic acids to accomplish the treatment of diabetes with a single molecule with multitarget action: activation of PPARγ, GLUT-4 and GPR40 and inhibition of AKR1B1 (Figure 1). Multitarget of polypharmacological concept can be defined as the modulation of several drug targets to achieve a desired therapeutic effect. This multitarget therapy may be an attractive option for the therapeutic treatment of diabetes that will not only control the glucose concentrations but also decrease complications linked to hyperglycemic conditions. Compounds **1**–**5** were designed based on the compounds GW9508 [16] and SHF-1 [17], maintaining the typical 4-point pharmacophore of synthetic GPR40 agonists [6], that mimic the fatty acid structure of endogenous agonists, such as docosahexaenoic acid (DHA, Figure 1): (I) an acidic group (carboxylate or surrogates); (II) a central *p*-substituted benzene; (III) a second lipophilic region and (IV) a flexible connector that joins regions (I) and (IV), and tolerating the structure to assume diverse conformations. The insulin-sensitizer targets PPARγ and GPR40 which are activated by long chain fatty acids and thiazolidine-2,4-diones are described to function as an integrated two-receptor signal transduction pathway [18] being sensitive to closely related pharmacophores, as represented by GW9508 and SHF-1 [17]. Then, it is reasonable to assume that also compounds **1**–**5** may act as agonists on these targets in a similar fashion. On the other hand, carboxylic acid derivatives comprise the majority of reported AKR1B1 inhibitors: epalrestat, tolrestat and zopolrestat. The other two chemical groups that function as inhibitors are: spirohydantoins (Sorbinil) and flavonoids such as quercetin [19]. Based on the relevance of the phenylpropanoic scaffold (Figure 1) in determining the inhibitory ability of zopolrestat towards AKR1B1, we expected that also compounds **1**–**5** may act as inhibitors of the enzyme. As a result of the multifactorial complexity of chronic diseases, the current therapeutic arsenal and the old-fashioned ‘‘one-molecule, one-target” model seem not so effective [20]. The design of new chemical entities (NCE) with two or more balancing bioactivities for the treatment of complex diseases would be very advantageous. Based on the above considerations, our work was focused on the design, preparation and evaluation of these novel multitarget molecules to be active against Diabetes. Although compound **1** was previously synthesized [21,22], in the current work the effectiveness as GPR40 agonist of this compound and of the accompanying molecules is extended and explored in vivo. A parallel investigation on the potential antidiabetic action of these compounds: the activation of PPARγ and GLUT-4 and the inhibition of AKR1B1, is also reported for first time. To our knowledge, this is the first report describing the potential of the simultaneous targeting of these four relevant processes linked to the diabetic pathology by a single molecule.

## 2. Results and Discussion

### 2.1. Chemical Synthesis

**Scheme 1:**
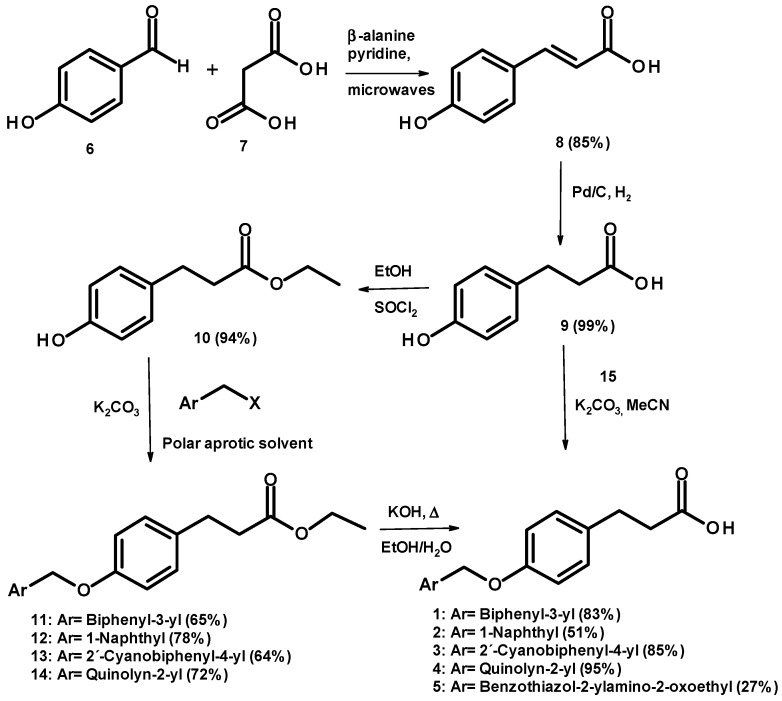
Synthesis of compounds **1**–**5**.

Compounds **1**–**5** were prepared starting from 4-hydroxybenzaldehyde (**6**), which was condensed with malonic acid (**7**), following by microwave Knoevenagel reaction and decarboxylation conditions to give compound **8**. This compound was hydrogenated under catalytic reduction with 10% Pd/C giving 3-(4-hydroxyphenyl)propanoic acid (**9**), which was reacted via esterification with thionyl chloride and ethanol as solvent, obtaining ethyl 3-(4-hydroxyphenyl)propanoate (**10**). Subsequently, **10** was reacted with the appropriately substituted methylaryl halide in polar aprotic solvents and potassium carbonate to give compounds **11**–**14**. All were treated separately by basic hydrolysis with potassium hydroxide, to give **1**–**4** (Scheme 1). Compound **5** was obtained by direct SN_2_ reaction between acid **9** and *N*-1,3-benzothiazol-2-yl-2-chloroacetamide (**15**). Compounds were recovered with modest yields and were purified by column chromatography or recrystallization (see experimental part). Their chemical structures were confirmed by spectroscopic (1D and 2D NMR, see supporting information) and spectrometric data, and their purity determined by microanalysis.

### 2.2. In Vitro GPR40 Activity

Compounds **1**–**5** were in vitro tested as GPR40 agonist. This activity was measured with calcium flux assay in GPR40-transfected HEK293 cells [2,23]. The preliminary screening results are summarized in Table 1. Linoleic acid (LA), one of the endogenous ligands for GPR40, was designated as positive control. Compounds were tested in quadruplicates at 100 μM. After preliminary screening, compounds **1**–**5** were further tested in a concentration-dependent manner (Table 1).

As we expected, the results indicated that compound **1** was the most potent agonist (EC_50_ = 0.075 μM), in accordance with the literature (EC_50_ = 0.260 μM) [21,22], followed by compounds **2** and **3,** with naphthyl and biphenylcarbonitrile substituents, respectively. All of them showed potencies in the submicromolar order. Compound **4**, with a quinolynyl substituent, provided a noteworthy reduction in agonistic action. Conversely, benzothiazole derivative **5** was inactive. Thus, compounds **2**–**5** were less active than previously reported **1**. On the other hand, it is noteworthy that the potency of compounds **1**–**3** is orders of magnitude greater than that obtained by endogenous fatty acid ligand LA.

### 2.3. In Vitro Aldose Reductase (AKR1B1) Inhibition

The in vitro AKR1B1 inhibitory activity of compounds **1**–**5** (Table 2) was evaluated by using a highly purified human recombinant enzyme preparation [24,25]. Sorbinil was employed as reference drug.

### 2.4. Relative Expression of PPARγ and GLUT-4

Compounds tested were shown interesting AKR1B1 inhibitors in the low micromolar IC_50_ values. Once again, compound **1**, previously identified as GPR40 agonist, was the most active inhibitor of the series, finding this new activity for this known compound. Compounds **2**, **3** and **5** were almost equally active. Compound **4** was the least active. None of them were more active than sorbinil, a well-known AKR1B1 inhibitor. As we described previously, AKR1B1 plays a key role in the progress of chronic diabetic complications, such as retinopathy, nephropathy, neuropathy and also cardiovascular risk. 

**Figure 2:**
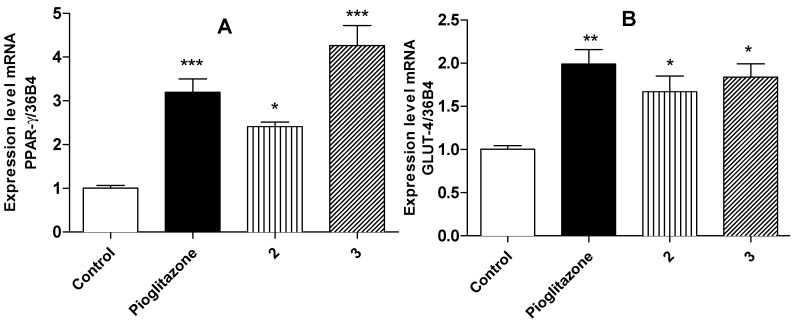
(**A**) Effect of compounds **2** and **3** on expression level of PPARγ; (**B**) Effect of compounds on expression level of GLUT-4. Results are mean ± SEM (*n* = 6)/*** *p* < 0.001; ** *p* < 0.01; * *p* < 0.05 compared with control group.

3T3-L1 fibroblasts were differentiated to the adipocyte phenotype in order to determine the action of compounds on PPARγ and GLUT-4 expression. Fibroblasts were treated during 24 h with compounds **1**–**5** and pioglitazone (10 μM) as reference drug and the mRNA expression level evaluated. Figure 2 shows that only compounds **2** and **3** enhance significantly the levels of relative expression for PPARγ (around three to four-times) and GLUT-4 mRNA's in the same way as pioglitazone. The activation of PPARγ could reduce the glucose levels in diabetic patients due to insulin resistance reduction. Our data also suggest that compounds **2** and **3** induces GLUT-4 expression in the same way than pioglitazone does. Several evidences indicate that increased levels of GLUT-4 expression in skeletal muscle are critical for the regulation of glucose homeostasis [7,17,26].

### 2.5. Molecular Docking Studies

Based on the in vitro biological assays and the preliminary enzyme inhibition evaluations, the most active compounds were selected to explain the experimental activities on these relevant targets. A preliminary molecular docking simulation was performed to assess the presumed binding mode of **1**–**5** into the receptors GPR40, PPARγ and the enzyme AKR1B1. A pilot in silico calculation was done using DIA-DB [27], a web server for the prediction of antidiabetic drugs via inverse virtual screening of the input molecules **1**–**5** against a set of 18 protein targets identified as key elements in diabetes, within which are included PPARγ, GPR40 and AKR1B1, among others [28]. Subsequently, a more specific and refined analysis was carried out for the most active compounds (**1**–**3**).

**Figure 3:**
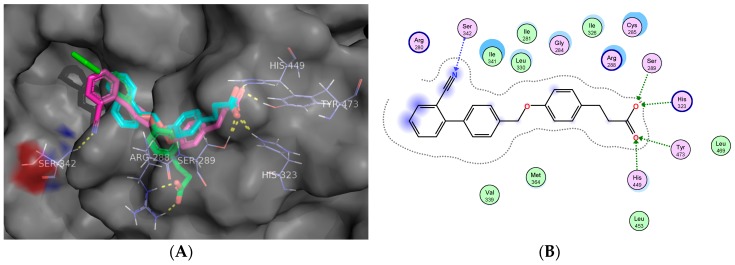
(**A**) 3D binding model of compounds **1**–**3** into the ligand binding site of PPARγ. Compounds are presented as stick models: **1** (green), **2** (cyan) and **3** (magenta), and aminoacids as lines. Dashed line signifies polar interactions; (**B**) 2D interaction map of the most active compound **3** and PPARγ.

Refined molecular docking reveals that compounds **2** and **3** internalize into the ligand binding site of PPARγ and interact by electrostatic and hydrogen bonds with Ser-289, His-323, His-449 and Tyr-473, all of them essential for the activation of this receptor. However, compound **3** (the most active in vitro) showed an additional interaction with Ser-342, characteristic of PPARγ partial agonists (Figure 3). Analyses of a huge number of crystallographic structures of the PPARχ ligand-binding site bound to an agonist have revealed that this isotype has two binding modes in a single pocket. These two binding modes correspond to full and partial agonists [29]. Side effects of glitazones, including weight gain, edema, congestive heart failure, and the recently reported increased risk of bone fracture are major undesired effects associated with the use of PPARγ full agonists [30]. On the other hand, partial agonists interact mainly through a hydrogen bond with Ser342. This interaction corresponds to several carboxylic ligands present in the majority of the PPARγ partial agonists that forms a hydrogen bond with the Ser342, such as showed by compound **3**.

**Figure 4:**
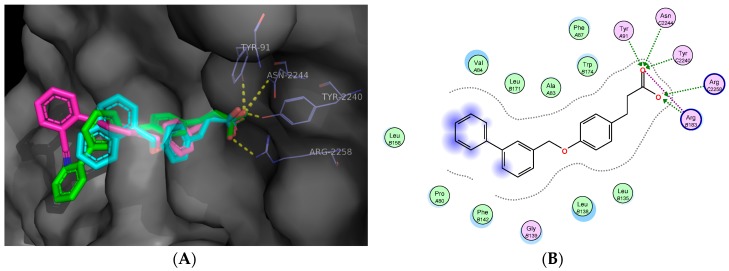
(**A**) 3D binding model of compounds **1**–**3** into the allosteric ligand binding site of GPR40. Compounds are presented as stick models: **1** (green), **2** (cyan) and **3** (magenta). (**B**) 2D interaction map of the most active compound **1** and GPR40.

For GPR40, binding poses depicted in Figure 4 suggest that the in vitro active compounds **1**, **2** and **3** interact through electrostatic bonds with residues of Arg-183 and Arg-2258, and by hydrogen bonds with Tyr-91, Asn-2244, Tyr-2240, all of them showed by well-known GPR40 allosteric agonists (such as TAK-875). On the other hand, the disposition of the biphenyl ring in **1**, which was the most potent in the in vitro screening, fits into the GPR40 ligand-binding-*pocket* better than the other compounds generating π-π interactions with Phe-142 (Figure 4B). The docking score for compound **1** was the highest (∆G = −10.63 kcal/mol), in comparison with compounds **2** and **3** (∆G = −10.31 and −9.96 kcal/mol, respectively).

**Figure 5:**
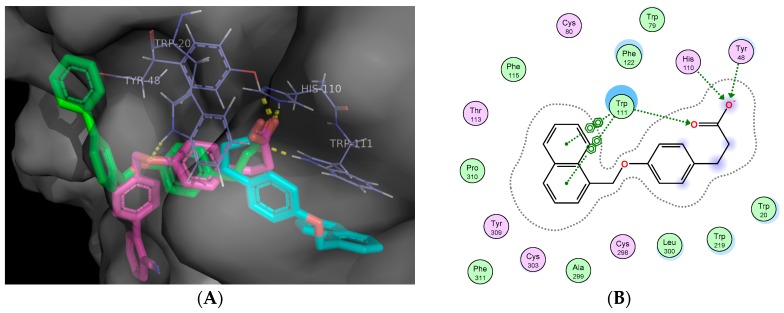
(**A**) 3D binding model of compounds **1**–**3** into the active site of Aldose reductase (AKR1B1). Compounds are presented as stick models: **1** (green), **2** (cyan) and **3** (magenta); (**B**) 2D interaction map of the second most active compound **2** and AKR1B1.

In the case of AKR1B1, solutions of molecular docking into the catalytic site of this enzyme show that acid moieties of compounds **1, 2** and **3** interact with Tyr-48, His-110 and Trp-111 showed in several currently inhibitors of this enzyme, such as zopolrestat and tolrestat. Also, the naphthyl ring of **2** conserves an interaction with Trp-111 through π-π stacking (Figure 5). All compounds showed moderate in vitro inhibition of this enzyme.

### 2.6. In Vivo Antidiabetic Effect of Compounds ***1***–***3***

**Figure 6:**
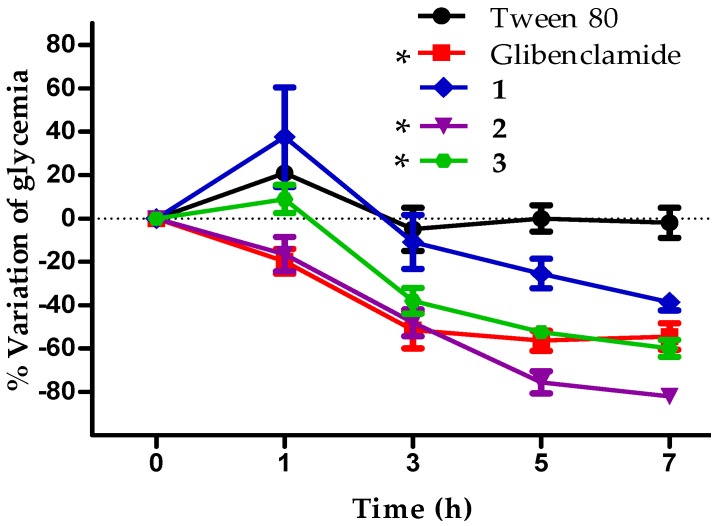
Effect of a single dose (100 mg/kg) of compounds **1**–**3** and glibenclamide (5 mg/kg) in STZ-NA induced diabetes mice model (intragastric, *n* = 6). * *p* < 0.05 versus Tween 80 (10%) group.

Compounds **1**–**3** were the most active against some of targets identified as key elements in diabetes in this work, and they were chosen in order to assess their in vivo activity over Streptozotocin (STZ) and Nicotinamide (NA) diabetic induced mice, a well-known non-insulin-dependent diabetes murine model [7,17]. Glibenclamide was used as a positive control, in order to ensure that the damage over β-cells was partial and mice pancreas still produces insulin, that responds to a secretagogue such as sulfonylurea drug used in this work. The antidiabetic activity of compounds **1**–**3** were determined via intragastric route of a single dose of 100 mg/kg (Figure 6).

In the in vivo assay, compound **1** exhibited potential differences in bioavailability that could explain the observed lack of in vivo activity for this compound**,** in comparison with the demonstrated activity of **2** and **3**. In particular, compounds **2** and **3** showed the best in vivo activity, compared with control group (Tween 80). Also, compound **2** producing a sustained decrease of blood glucose levels, close to 80% less, 7 h after compound administration (Figure 6).

This compound showed a statistically significant hypoglycemic effect like glibenclamide during the time that the experiment lasted. The glucose lowering effect of compound **3** was increasing after the first hour of administration (−20% at 3 h, −50% at 5 h, and −60% at 7 h). Besides, statistically significant difference is detected between **3** and tween 80 group, and the maximum effect was detected in the last hours of post-intragastric administration (Figure 6).

### 2.7. Off-Target Toxicity Predictions

With the aim of anticipating potential off-targets affinity and toxicity issues of the most active compounds **1**–**3**, a virtual prediction of safety profiles was accomplished. The toxicity parameters of **1**–**3**, pioglitazone, sorbinil and glibenclamide (in vitro and in vivo antidiabetic reference drugs) were calculated through the ACD/ToxSuite software, v. 2.95 (Table 3).

The in silico calculation of inhibition for the three main isoforms of CYP450 for compounds **1**–**3** were comparable to pioglitazone, sorbinil and glibenclamide at relevant clinical concentrations (<10 μM), showing low probabilities of drug-drug interactions and undesirable adverse effects [31]. Besides, several lipophilic compounds are associated with cardiovascular risks due to human ether-a-go-go related gene (hERG) channel blockade [7,32]. Compounds **1**–**3**, contemplated as polypharmacological molecules, showed low prediction of hERG channel blockage at clinically relevant concentrations (*K_i_* < 10 μM), being considered as potentially non-cardiotoxic compounds. In the calculation of acute toxicity, compounds **1**–**3** demonstrated similar or even high predicted LD_50_ than pioglitazone, sorbinil and glibenclamide by two different administration routes, being predicted less toxic than these common antidiabetic drugs used as reference in this work.

Several examples of polypharmacology have been reported elsewhere [33,34]. Actually, some multikinase inhibitors used in cancer treatment and all marketed antipsychotics are polypharmacological drugs, even recently approved compounds. Multitargeting concept explains the efficacy of these drugs by their modulation of a network of disease relevant targets correcting a pathological imbalance [33].

## 3. Materials and Methods

### 3.1. Chemistry

All compounds were purchased from Sigma-Aldrich (Saint Louis, MO, USA). Melting points were recorded using an EZ-Melt MPA120 automated apparatus from Stanford Research Systems (Sunnyvale, CA, USA). Reactions were monitored by TLC on 0.2 mm precoated silica gel 60 F_254_ plates (Merck, Darmstadt, Germany). ^1^H-NMR spectra were determined on a Varian Oxford instrument (Palo Alto, CA, USA) operating at 600 MHz (^1^H-NMR) and 150 MHz (^13^C-NMR) instruments. Chemical shifts are given in ppm in DMSO-*d*_6_ and CDCl_3_ as deuterated solvents. Mass spectrometry were determined on a JMS-700 spectrometer (JEOL, Tokyo, Japan) operating in electronic impact mode.

### 3.2. General Procedure for the Synthesis of Compounds ***1***–***4***

Ethyl esters **11**–**14** (0.70 mmol) were treated with a mixture of EtOH/H_2_O (1:1, *v*/*v*) and KOH (2 equiv.). The mixture was stirred at reflux for 3.5–5 h. Subsequently, the ethanol was removed under vacuum and HCl solution (10%, *v*/*v*) was added until reach pH 5, to obtain a precipitate which was filtered and washed with cold water to give a solid. The crude products were recrystallized from ethanol or methanol affording title compounds.

*3-[4-(Biphenyl-3-ylmethoxy)phenyl]propanoic acid* (**1**). Yield 83%, pearly flakes m.p. 123.7–126 °C; ^1^H-NMR (DMSO-*d*_6_) δ: 2.43 (t, 2H, CH_2_), 2.74 (t, 2H, CH_2_), 5.13 (s, 2H, CH_2_), 6.94 (d, 2H, H-3A, H-5A, *J* = 7.74 Hz), 7.14 (d, 2H, H-2A, H-6A, *J* = 8.04 Hz), 7.4 (t, 1H, H-4C), 7.43 (d, 1H, H-4B, *J* = 7.2 Hz), 7.48–7.46 (m, 3H, H-6B, H-3C, H-5C), 7.61 (t, 1H, H-5B, *J* = 7.5 6Hz), 7.66 (d, 2H, H-2C, H-6C, *J* = 8.22 Hz), 7.72 (s, 1H, H-2C) ppm. ^13^C-NMR (DMSO-*d*_6_) δ: 30.4 (CH_2_), 36.9 (CH_2_), 69.6 (O-CH_2_), 115.1 (C-3A, C-5A), 126.4 (C-6B), 126.6 (C-4B), 127.1 (C-2C, C-6C), 128 (C-4C), 129.4 (C-3C, C-5C), 129.5 (C-5B), 129.7 (C-2A, C-6A), 134 (C-1A), 138.5 (C-1C), 140.4 (C-1B), 140.8 (C-3B), 157.0 (C-4A), 174.7 (C=O) ppm. MS/EI: *m*/*z* (% int. rel). 353 (M + Na), 178 (M + Na − 175) 100%. Anal. Calcd. for C_22_H_20_O_3_: C 79.50, H 6.06; Found C 79.54, H 6.04.

*3-[4-(1-Naphthylmethoxy)phenyl]propanoic acid* (**2**). Yield 51%, white powder m.p. 124.8–127.8 °C; ^1^H-NMR (DMSO-*d*_6_) δ: 2.49 (t, 2H, CH_2_), 2.76 (t, 2H, CH_2_), 5.50 (s, 2H, CH_2_), 7.0 (d, 2H, H-3A, H-5A, *J* = 8.46 Hz), 7.16 (d, 2H, H-2A, H-6A, *J* = 8.46 Hz), 7.51 (t, 1H, H-3′, *J* = 7.56 Hz), 7.58–7.55 (m, 2H, H-6′, H-7′), 7.66 (d, 1H, *J* = 6.9 Hz), 7.92 (d, 1H, *J* = 8.52 Hz), 7.97 (d, 1H, *J* = 7.86 Hz), 8.08 (d, 1H, *J* = 7.97 Hz). δ: ^13^C-NMR (DMSO-*d*_6_) δ: 30.0 (CH_2_), 36.1 (CH_2_), 68.2 (CH_2_), 115.1 (C-3A, C-5A), 124.3 (C-8′), 125.8 (C-3′), 126.4 (C-6′), 126.9 (C-7′), 127.03 (C-2′). 128.9 (C-5′), 129.0 (C-4′), 129.7 (C-2A, C-6A), 131.6 (C-8a), 133.1 (C-1A), 133.6 (C-4a), 133.7 (C-1′), 157.2 (C-4A, 174.3 (C=O) ppm. MS/EI: *m*/*z* (% int. rel.). 306 (M^+^), 141(M − 165)100%. Anal. Calcd. for C_20_H_18_O_3_: C 78.41, H 5.92; Found C 78.43, H 5.91.

*3-{4-[(2′-Cyanobiphenyl-4-yl)methoxy]phenyl} propanoic acid* (**3**). Yield 85%, pearly flakes m.p. 174–176.9; ^1^H-NMR (CDCl_3_) δ: 2.66 (t, 2H, CH_2_), 2.91 (t, 2H, CH_2_), 5.11 (s, 2H, CH_2_), 6.93 (d, 2H, H-3A, H-5A, *J* = 8.52 Hz), 7.15 (d, 2H, H-2A, H-6A, *J* = 8.46 Hz), 7.44 (t, 1H, H-4C, *J* = 7.68 Hz), 7.51 (d, 1H, H-6C, *J* = 7.86 Hz), 7.55 (2H, H-3B, H-5B, *J* = 8.22 Hz), 7.58 (2H, H-2B, H-6B, *J* = 8.16 Hz), 7.63 (t, 1H, H-5C, *J* = 7.62 Hz), 7.76 (d, 1H, H-3C, *J* = 7.74 Hz), 12.10 (s, 1H, OH) ppm. ^13^C-NMR (CDCl_3_) δ: 29.8 (CH_2_), 35.9 (CH_2_), 69.6 (CH_2_), 111.3 (C-2C), 118.6 (CN), 114.9 (C-3A, C-5A), 127.6 (C-4C), 127.7 (C-3B, C-5B), 129 (C-2B, C-6B), 129.3 (C-2A, C-6A), 130.0 (C-6C), 132.8 (C-5C), 133.8 (C-3C), 137.7 (C-4B), 137.8 (C-1B), 145.1 (C-1C), 157.2 (C-4A), 178.7 (C=O) ppm. MS/EI: *m*/*z* (% int. rel). 355 (M^+^), 192 (M − 165) 100%. Anal. Calcd. for C_23_H_19_NO_3_: C 77.29, H 5.36, N 3.92; Found C 77.31, H 5.34, N 3.94.

*3-[4-(Quinolin-2-ylmethoxy)phenyl]propanoic acid* (**4**). Yield 95%, yellow crystals m.p. 125.8–128.4 °C; ^1^H-NMR (DMSO-*d*_6_) δ: 2.49 (t, 2H, CH_2_), 2.74 (t, 2H, CH_2_), 5.33 (s, 2H, CH_2_), 6.97 (d, 2H, H-3A, H-5A, *J* = 8.82 Hz), 7.15 (d, 2H, H-2A, H-6a, *J* = 8.34 Hz), 7.62 (t, 1H, H-6′, *J* = 7.8, *J* = 7.14 Hz), 7.66 (d, 1H, H-3′, *J* = 8.4 Hz), 7.79 (t, 1H, H-7′, *J* = 6.96, *J* = 8.34 Hz), 7.99, d, 1H, H-5′, *J* = 8.16 Hz), 8.02 (d, 1H, H-4′, *J* = 8.46 Hz), 8.41, d, 1H, H-8′, *J* = 8.52 Hz), 12.1 (s, 1H, OH) ppm. ^13^C-NMR (DMSO-*d*_6_) δ: 29.9 (CH_2_), 35.9 (CH_2_), 71.2 (CH_2_), 115.1 (C-3A, C-5A), 119.9 (C-3′), 127.01 (C-6′), 128.4 (C-4a), 129.0 (C-7′), 129.8 (C-2A, C-6A), 130.3 (C-5′), 133.8 (C-1A), 137.5 (C-4′), 147.4 (C-8a), 156.9 (C-4A), 158.2 (C-2′), 174.2 (C=O) ppm. MS/EI: *m*/*z* (% int. rel). 307 (M^+^) 100%, 142 (M − 165) 75%. Anal. Calcd. for C_19_H_17_NO_3_: C 74.25, H 5.58, N 4.56; Found C 74.20, H 5.52, N 4.56.

### 3.3. Synthesis of 3-{4-[2-(1,3-Benzothiazol-2-ylamino)-2 oxoethoxy]phenyl}propanoic Acid *(**5**)*

A stirred mixture of *N*-1,3-benzothiazol-2-yl-2-chloroacetamide **15** (0.0006 mol), 3-(4-hydroxyphenyl)propanoic acid **9** (1 equiv.), potassium carbonate (3 equiv.), potassium iodide (10% mol) in 3 mL of acetonitrile was refluxed. The reaction solvent was dried and the mixture was basified at pH = 3 with HCl 10% (*v*/*v*). The solid was filtered and dried to give compound **5**. Yield 27%, low melting point solid; ^1^H-NMR (DMSO-*d*_6_) δ: 2.41 (t, CH_2_, H-2), 2.70 (t, CH_2_, H-3), 4.78 (s, CH_2_, H-4), 7.00 (t, H-5A, *J* = 7.74 Hz), 7.20 (t, H-6A, *J* = 7.68 Hz), 7.32 (d, H-2, H-6B, *J* = 8.64 Hz), 7.45 (s, H-3B, H-5B), 7.64 (d, H-4A, H-7A, *J* = 7.80 Hz). ^13^C-NMR (DMSO-*d*_6_) δ: 19.24 (C-3), 35.09 (C-2), 65.00 (C-4), 119.47 (C-4B), 120.16 (C-1B), 122.86 (C-4A, C-7A), 125.98 (C-3B, C-5B), 125.99 (C-2B, C-6B), 130.55 (C-5A, C-6A), 134.17 (C-7A), 134.29 (C-3A), 136.06 (C-2 A), 157.95 (C-5), 171.56 (C-1); MS-ESI: *m*/*z* 357 (M + H^+^). Anal. Calcd. for C_18_H_16_N_2_O_4_S: C 60.66, H 4.53, N 7.86; Found C 60.71, H 4.54, N 7.84.

### 3.4. Synthesis of (2E)-3-(4-Hydroxyphenyl)acrylic Acid *(**8**)*

4-Hydroxybenzaldehyde **6** (0.25 g, 2.04 mmol), β-alanine (0.1 equiv, 0.20 mmol) and malonic acid **7** (0.21g, 1.1 equiv, 2.06 mmol) were suspended in pyridine (5 mL), following Knoevenagel reaction with subsequent decarboxylation. The flask was shaken well and heated under microwave irradiation system (Discovery Microwave System apparatus, 2450 MHz, 300 W, CEM, Matthews, NC, USA) for 10 min at 70 °C. After irradiation, the mixture was verted in cold water and acidified until pH 5. The precipitate was collected by filtration, washed with water, dried and recrystallized from ethanol. Yield 80%, yellow crystals m.p. 214 °C (dec); ^1^H-NMR (DMSO-*d*_6_) δ: 6.35 (d, 1H, =CH *trans*, *J* = 16 Hz), 6.86 (d, 2H, H-3′, H-5′, *J* = 8.4 Hz), 7.55 (d, 2H, H-2′, H-6′, *J* = 8.4 Hz), 7.58 (d, 1H, =CH *trans*, *J* = 16 Hz), 8.87 (bs, 2H, OH, COOH) ppm.^13^C-NMR (DMSO-*d*_6_) δ: 115.4 (C-2), 116.2 (C-3′, C-5′), 126.1 (C-1′), 130.4 (C-2′, C-6′), 145.3 (C-3), 158.3 (C-4′), 168.6 (C=O) ppm. MS/EI: *m*/*z* (% int. rel). 164 (M^+^).

### 3.5. Synthesis of 3-(4-Hydroxyphenyl)propanoic Acid *(**9**)*

A mixture of acid **8** (0.2 g, 1.20 mmol) and Pd/C 10% (0.02 g) in ethanol (40 mL) was hidrogenated at 60 lb/in^2^ in a hydrogenation apparatus for 30 min. After filtration and evaporation of the solvent uder reduce pressure a yellow oil was obtained. After cooling, a solid compound was obtained which purificated by recrystallyzation in ethanol. Yield 99%, yellow crystals m.p. 129–131 °C; ^1^H-NMR (DMSO-*d*_6_) δ:2.45 (t, 2H, CH_2_), 2.72 (t, 2H, CH_2_), 6.65 (d, 2H, H-3′, H-5′, *J* = 8.01 Hz), 7.05 (d, 2H, H-2′, H-6′, *J* = 8.02 Hz), 9.54 (bs, 2H, OH, COOH) ppm.^13^C-NMR (DMSO-*d*_6_) δ: 30.1 (CH_2_), 36.9 (CH_2_), 115.5 (C-3′, C-5′), 129.5 (C-2′, C-6′), 131.8 (C-1′), 155.5 (C-4′), 175.1 (C=O) ppm. MS/EI: *m*/*z* (% int. rel). 166 (M^+^).

### 3.6. Synthesis of Ethyl 3-(4-hydroxyphenyl)propanoate *(**10**)*

To a stirred solution of acid **9** (3 g, 18 mmol) in 30 mL of ethanol, under ice-cooling, was added thionyl chloride (1.1 eq, 1.44 mL, 19 mmol) dropwise over 20 min. After stirring the reaction mixture for 3 h at room temperature, methanol is distilled out and 25 mL of water is added. The separated ester is extracted with ethyl acetate and washed with 10 mL of saturated sodium bicarbonate solution. Evaporation of the ethyl acetate gave the ester in pure form. Yield 94%, yellow liquid; ^1^H-NMR (CDCl_3_) δ: 1.22 (d, 3H, CH_3_), 2.59 (t, 2H, CH_2_), 2.87 (t, 2H, CH_2_), 4.12, q, 2H, CH_2_), 6.74 (d, 2H, 2.09, 6.48, H-3A-H-5A), 7.03 (d, 2H, 6.54, H-2A, H-6A). ^13^C-NMR (CDCl_3_) δ: 14.2 (CH_3_), 30.2 (CH_2_), 36.3 (CH_2_), 60.6 (CH_2_), 115.4 (C-3A, C-5A), 129.4 (C-2A, C-6A), 154.3 (C-4A), 173.6 (C=O). MS/EI: *m*/*z* (% int. rel). 194 (M^+^) 49%, 142 (M − 74) 100%.

### 3.7. General Method for the Preparation of Compounds ***11***–***14***

A solution of ethyl 3-(4-hydroxyphenyl)propanoate **10** (0.5 g, 2.5 mmol) and K_2_CO_3_ (0.71 g, 5.1 mmol, 2 equiv) in appropriate polar aprotic solvent (5 mL acetonitrile or acetone) was heated at 40 °C for 30 min. Adequately substituted methylaryl halides (1.1 equiv.) were added dropwise. The mixture was heated to reflux for 8 h. Solvent was evaporated in vacuo. A mixture of ice-water (10 mL) was added and stirred for 15 min. The emulsion formed was extracted with ethyl acetate (3 × 15 mL). The solvent was removed in vacuo to give an oily product, which were purified by column chromatography.

*Ethyl 3-[4-(biphenyl-3-ylmethoxy)phenyl] propanoate* (**11**). Yield 65%, colorless liquid; ^1^H-NMR (CDCl_3_) δ: 1.21 (t, 3H, CH_3_), 2.58 (t, 2H, CH_2_), 2.88 (t, 2H, CH_2_), 4.11 (c, 2H, CH_2_′), 5.08 (s, 2H, CH_2_), 6.92 (d, 2H, *J* = 7.8, H-3A, H-5A), 7.11 (d, 2H, *J* = 8.1, H-2A, H-6A), 7.34 (t, 1H, *J* = 7.56, H-5B), 7.39 (d, 1H, 7.44, H-6B), 7.43–7.41 (m, 3H, H-3C, H-4C, H-5C), 7.54 (d, 1H, *J* = 7.68, H-4B), 7.58, (d, 2H, *J* = 7.68, H-2C, H-6C), 7.64 (s, 1H, H-2B) ppm. ^13^C-NMR (CDCl_3_) δ: 14.2 (CH_3_), 30.1 (CH_2_), 36.2 (CH_2_), 60.3 (CH_2_′), 70.1 (CH_2_), 114.9 (C-3A, C-5A), 126.2 (C-4B), 126.3 (C-2B), 126.7 (C-BB), 127.2 (C-2C, C-6C), 127.4 (C-5B), 128.9 (C-3C, C-5C), 129 (C-4C), 129.3 (C-2A, C-6A), 133.0 (C-1A), 137.7 (C-1C), 140.9 (C-1B), 141.5 (C-3B), 157.2 (C-4A), 172.9 (C=O) ppm. MS/EI: *m*/*z* (% int. rel). 360(M^+^) 31%, 167(M − 193) 100%.

*Ethyl 3-[4-(1-naphthylmethoxy)phenyl]propanoate* (**12**). Yield 78%, colorless liquid; ^1^H-NMR (CDCl_3_) δ:1.23 (t, 3H, CH_3_), 2.59 (t, 2H, CH_2_) 2.90 (t, 2H, CH_2_) 4.12 (c, 2H, CH_2_′), 5.44 (s, 2H, CH_2_), 6.97 (d, 2H, 7.14, H-3A, H-5A) 7.14 (d, 2H, *J* = 7.68, H-2A, H-6A) 7.44 (t, 1H, *J* = 8.22, *J* = 8.28, H-3′) 7.52–7.49 (m, 2H, H-6′, H-7′) 7.57 (d, 1H, *J* = 6.9, H-2′) 7.83 (d, 1H, *J* = 8.22, H-4′) 7.85 (d, 1H, *J* = 7.5, H-5′) 8.03 (d, *J* = 7.86, H-8′) ppm. ^13^C-NMR (CDCl_3_) δ: 14.2 (CH_3_), 30.2 (CH_2_), 36.2 (CH_2_), 60.4 (CH_2_′), 68.7 (CH_2_), 114.9 (C-3A, C-5A), 123.7 (C-8′), 125.3 (C-3′), 125.9 (C-6′), 126.5 (C-7′), 128.7 (C-2′), 128.9 (C-5′), 129.3 (C-2A, C-6A), 131.5 (C-4′), 132.4 (C-8a), 133.1 (C-4a), 133.7 (C-1′), 157.4 (C-4a), 172.9 (C=O) ppm. MS/EI: *m*/*z* (% int. rel). 334 (M^+^) 11%, 141(M − 193) 100%.

*Ethyl 3-{4-[(2′-cyanobiphenyl-4-yl)methoxy] phenyl}propanoate* (**13**). Yield 64%, white crystals; ^1^H-NMR (DMSO-*d*_6_) δ: 1.14 (t, 3H, CH_3_), 2.56 (t, 2H, CH_2_) 2.78 (t, 2H, CH_2_) 4.03, (q, 2H, CH_2_′), 5.16 (s, 2H, CH_2_) 6.96 (d, 2H, *J* = 8.04, H-3A, H-5A) 7.15 (d, 2H, *J* = 8.04, H-2A, H-6A), 7.64-7.60 (m, 6H, H-2A, H-3A, H-5A, H-6A, H-4C, H-6C), 7.79 (t, 1H, *J* = 7.56, H-5C) 7.95 (d, 1H, *J* = 7.68, H-3C) ppm. ^13^C-NMR (DMSO-*d*_6_) δ:14.5 (CH_3_), 29.1 (CH_2_), 35.8 (CH_2_), 60.2 (CH_2_′), 69.1 (CH_2_), 110.6 (C-2C), 115 (C-3A, C-5A), 119 (CN), 128.7 (C-4C), 129.2 (C-2B, C-6B), 129.7 (C-2A, C-6A), 130.5 (C-6C), 133.2 (C-1A), 134 (C-5C), 134.3 (C-3C), 137.7 (C-4B), 138.3 (C-1B), 144.7 (C-1C), 157.2 (C-4A), 172.5 (C=O) ppm. MS/EI: *m*/*z* (% int. rel). 385 (M^+^) 11%, 192(M − 193) 100%.

*Ethyl 3-[4-(quinolin-2-ylmethoxy)phenyl]propanoate* (**14**). Yield 72%, yellow liquid; ^1^H-NMR (CDCl_3_) δ: 1.47 (t, 3H, CH_3_), 2.50 (t, 2H, CH_2_), 2.75 (t, 2H, CH_2_), 4.01 (c, 2H, CH_2_), 5.32 (s, 2H, CH_2_), 6.96 (d, 2H, *J* = 6.66, *J* = 8.67, H-3A, H-5A), 7.13 (d, 2H, *J* = 8.58, H-2A, H-6A), 7.62, (d, 1H, *J* = 7.35, H-6′), 7.65 (d, 1H, *J* = 8.49, H-3′), 7.79 (dd, 1H, *J* = 8.28, H-7′), 7.97 (dd, 1H, *J* = 8.85, H-5′), 8.01 (dd, 1H, *J* = 8.64, H-4′), 8.39 (d, 1H, 8.55, H-8′). ^13^C-NMR (CDCl_3_) δ: 14.0 (CH_3_), 29.4 (CH_2_), 35.4 (CH_2_), 59.7 (CH_2_′), 70.7 (CH_2_), 114.6 (C-3A, C-5A), 119.5 (C-3′), 127.1 (C-6′), 128.1 (C-4′), 128.5 (C-4a), 129.1 (C-7′), 129.8 (C-2A, C-6A), 130.5 (C-5′), 132.9 (C-1A), 137.9 (C-8′), 146.9 (C-8a), 158.7 (C-2′), 172.1 (C=O) ppm. MS/EI: *m*/*z* (% int. rel). 335 (M^+^) 10%. 

### 3.8. Synthesis of N-1,3-Benzothiazol-2-yl-2-chloroacetamide *(**15**)*

A mixture of 2-amino-1,3-benzothiazole (0.100 g), sodium bicarbonate (3 equiv) in acetone (10 mL), was stirred at 5 °C. After 30 min, chloroacetyl chloride (1.5 equiv.) was added dropwise using a drop funnel apparatus and stirred at room temperature for 8 h. Once the reaction was completed, the solvent was removed in vacuo, and the white precipitated was washed with cold water. Yield: 90%, m.p.: 156.5–159.3 °C; ^1^H-NMR (CDCl_3_) δ: 4.32 (s, CH_2_), 7.36 (t, H-6, *J* = 8.10 Hz), 7.48 (t, H-5, *J* = 8.28 Hz), 7.84 (q, H-4, H-7, *J* = 7.92 Hz); MS-EI: *m*/*z* 225.75 (M^+^) 40%, 149.61(M − 77) 100%.

### 3.9. Biological Assays

#### 3.9.1. GPR40 Agonistic Activities of Compounds **1**–**5**

This bioassay was previously described [2]. Briefly: About 4 × 10^4^ h GPR40/HEK293 per well were seeded in 96-well plate. After culturing for 24 h, cells were loaded with 2 μmol/L Fluo-4 AM in Hanks’ balanced salt solution at 37 °C for 40 min. After thorough washing with 50 μL assay buffer, 25 μL assay buffer containing compound was dispensed into the well, using a FlexStation III microplate reader and intracellular calcium change was recorded with an excitation wavelength of 485 nm and emission wavelength of 525 nm.

#### 3.9.2. Aldose Reductase (AKR1B1) Inhibition Assay

Human recombinant AKR1B1 was purified by electrophoretic homogeneity as previously described [24]. The enzyme activity was corroborated at 37 °C following the decrease of absorbance at 340 nm. The assay mixture (final volume of 0.7 mL) contained 0.25 M Na_3_PO_4_ buffer (pH 6.8), 4.67 mM d,l-glyceraldehyde, 0.11 mM NADPH, 0.38 M (NH_4_)_2_SO_4_ and 0.5 mM EDTA. Compounds **1**–**5**, tested as AKR1B1 inhibitors were dissolved at appropriate concentrations in a mixture DMSO/methanol (40:60, *v*/*v*) and added to the assay mixture containing 5 mU of AKR1B1; the concentrations of DMSO and methanol in all the assays were kept constant at 0.2 and 0.3% (*v*/*v*), respectively. IC_50_ values was determined by nonlinear regression analysis. For each curve, five different concentrations of compounds were analyzed [25].

#### 3.9.3. In Vitro PPARγ and GLUT-4 Assay

Both assays were achieved as described elsewhere [7,17,35,36,37]. Briefly: 3T3-L1 fibroblasts (9 × 10^5^ cells per well) were cultured in 6-well plates in Dulbecco’s modified Eagle’s medium. After 2 days of confluence, the cells were differentiated to the adipocyte phenotype with 0.5 mM 3-isobutyl-1-methylxanthine, 0.25 μM dexamethasone acetate, and 0.8 μM insulin, for 48 h, followed by insulin alone for other 48 h. To determine the action of compounds on PPARγ and GLUT-4 expression, the cells were treated 24 h. RNA was isolated from cultured cells and two μg of total RNA were reverse-transcripted in a thermocycler. The enzyme was inactivated and finally samples were cooled. Then 1/10 volume of each reverse-transcripted reaction was amplified with SYBR Green master mix containing 0.5 mM of customized primers for PPAR-γ and GLUT-4. PCR was conducted and the threshold cycles (Ct) were measured in separate tubes and in duplicate. The ΔCt values were calculated in every sample for each gene of interest. Relative changes in the expression level of one specific gene (ΔΔCt) were calculated [37].

### 3.10. In Vivo Assay

CD1 mice (20–25 g) were housed in groups of six (*n* = 6) under laboratory conditions. Before experimentation, all animals were fasted for 16 h with water ad libitum. All animal procedures were conducted in accordance with the Mexican Federal Regulations for Animal Experimentation and Care (SAGARPA, NOM-062-ZOO-1999), based on US National Institutes of Health Publication #85-23, revised 1985. Non-insulin-dependent diabetes mice model was induced in overnight fasted mice by a single i.p. injection of 100 mg/kg STZ, 15 min before the i.p. administration of 40 mg/kg of NA. Hyperglycaemia was established by the higher plasma glucose concentration, determined by glucometer after 2 weeks. The animals with glycaemia higher than 200 mg/dL were selected for the assay [38,39,40,41]. The diabetic mice were separated into three groups of six animals each (*n* = 6). Mice of experimental groups were administered with a suspension of the molecules **1**–**3** (100 mg/kg, prepared in 10% (*v*/*v*) tween 80). Control group mice were similarly treated with 10% tween 80. The hypoglycemic reference drug glibenclamide was used as positive control (5 mg/kg). The samples of blood were collected from the caudal vein at 0, 1, 3, 5 and 7 h after administration of each compound. Blood glucose levels were estimated using a commercial glucometer [41,42,43]. The percentage variation of glycemia for each group was calculated. Values are expressed as mean ± S.E.M. Statistical significance was estimated by ANOVA.

### 3.11. In Silico Docking Studies

The crystal structures of PPARγ, GPR40 and aldose reductase were recovered from the Protein Data Bank (PDB) with the ID: 1I7I, 4PHU and 1Z3N respectively. Docking calculations were performed using AutoDock, version 4.2. The program performs several runs (100) in each docking experiment. Each run provides one predicted binding mode. All water molecules and every co-crystall ligands were removed from the crystallographic structure and all hydrogen atoms were added. For all ligands and proteins, Gasteiger charges were assigned and non-polar hydrogen atoms were merged. All torsions could rotate during docking. The auxiliary program AutoGrid generated the grid maps. Each grid was centered at the crystallographic coordinates of the crystallographic compound with dimensions of 60 × 60 × 60 Å with points separated by 0.375 Å. After molecular docking, all solutions were clustered into groups with RMSD < 2.0 Å. Discovery Studio version 3.5, Pymol version 1.7.4 and MOE [44] were used for visualization.

#### Docking Validation

The docking protocols were validated by re-docking of cocrystal ligands into the active site of the structure of each protein target: PPARγ (Tesaglitazar), GPR40 (TAK-875) and aldose reductase (Lidorestat). The RMSD obtained in both cases was less than 2.0 Å in all cases. This value specifies that the parameters for docking simulations agree in reproducing the orientation and the conformation in the X-ray crystal coordinates of enzyme and receptors.

## 4. Conclusions

Five phenylpropanoic acid derivatives active on different relevant targets involved in the control of glucose level and in the cell damage linked to hyperglycaemic conditions, are presented as promising antidiabetic compounds. In fact, compounds **1** to **3** are able with different effectiveness to combine a significantly increase in the mRNA expression of PPARγ, GLUT-4, with a GPR40 agonist action and a well detectable inhibition of the AKR1B1 activity. Compounds **2** and **3** also revealed their antidiabetic effectiveness in vivo, showing a robust hypoglycemic efficacy similar to glibenclamide. The in vitro multitargeting profile and the in vivo hypoglycemic efficacy make some of them suitable leads in developing new chemical entities with polypharmacological ability for treatment of diabetes and its complications. To our knowledge, this is the first report describing a multiple combined action of a single molecule facing towards these four relevant targets involved into cellular glucose handling.

## Figures and Tables

**Table 1:** Preliminary screening (Efficacy, maximal response) and GPR40 agonistic activities (Potency, EC_50_) of compounds **1**–**5** in calcium assay for hGPR40/Gα16/HEK293.

Comp.	Maximal Response % (100 μM)	EC_50_ (μM) ^a^
**1**	98 ± 10	0.075 ± 0.02
**2**	103 ± 3	0.648 ± 0.10
**3**	105 ± 15	0.797 ± 0.17
**4**	84	≈100
**5**	9.9	>100
**LA**	100	8.30 ± 1.7

^a^ Means of two experiments.

**Table 2:** Inhibition of human recombinant AKR1B1 by compounds **1**–**5**.

Comp.	IC_50_ (μM) ^a^
**1**	8.9 (6.5–12.1)
**2**	17.4 (14.3–21.2)
**3**	21.0 (12.8–34.3)
**4**	31.7 (22.3–44.9)
**5**	23.3 (19.0–25.5)
**Sorbinil**	1.2 (0.9–1.5)

^a^ 95% confidence intervals are reported in brackets.

**Table 3:** Toxicity profiles predicted for compounds **1**–**3**, pioglitazone, sorbinil and glibenclamide.

Compd	LD_50_ (mg/kg)				Probability of Inhibition (IC_50_ or *K_i_* < 10 μM)			
	Mouse		Rat		CYP450 Isoform			hERG
	i.p.	p.o.	i.p.	p.o.	3A4	2D6	1A2	
**1**	320	1400	560	3800	0.08	0.05	0.17	0.10
**2**	530	2000	460	2700	0.07	0.05	0.15	0.04
**3**	580	1990	1200	4000	0.07	0.02	0.04	0.13
**Pioglitazone**	440	1900	400	1100	0.22	0.03	0.08	0.21
**Sorbinil**	420	680	190	1600	0.02	0.02	0.01	0.10
**Glibenclamide**	980	1900	500	2400	0.20	0.08	0.01	0.60

## Supplementary Materials

Spectra are available online.
