# Supplementary material for: Design, Synthesis and in Combo Antidiabetic Bioevaluation of Multitarget Phenylpropanoic Acids
Source: Molecules. 2018 Feb 6;23(2):340. doi: 10.3390/molecules23020340 (PMC6017591; doi:10.3390/molecules23020340)
Supplement: Supplementary file 1 [file molecules-23-00340-s001.pdf]

Supporting Information

Design, Synthesis and in combo Antidiabetic Bioevaluation of Multitarget Phenylpropanoic Acids †

Blanca Colín-Lozano [1], Samuel Estrada-Soto [1], Fabiola Chávez-Silva [1], Abraham Gutiérrez-Hernández [1], Litzia Cerón-Romero [1], Abraham Giacoman-Martínez [2], Julio Cesar Almanza-Pérez [2], Emanuel Hernández-Núñez [3], Zhilong Wang [4], Xin Xie [4], Mario Cappiello [5], Francesco Balestri [5], Umberto Mura [5] and Gabriel Navarrete-Vazquez [1,*]

[1] Facultad de Farmacia, Universidad Autónoma del Estado de Morelos, Cuernavaca, Morelos 62209, Mexico; clbi_ff@uaem.mx (B.C.-L.); enoch@uaem.mx (S.E.-S.); facasy@gmail.com (F.C.-S.); ghaa_ff@uaem.mx (A.G.-H.); crlc_ff@uaem.mx (L.C.-R.)

[2] Laboratorio de Farmacología, Departamento de Ciencias de la Salud, Universidad Autónoma Metropolitana Iztapalapa, Ciudad de México 09340, Mexico; agmfest@hotmail.com (A.G.-M.); j.almanza.perez@gmail.com (J.C.A.-P.)

[3] Cátedra CONACyT, Departamento de Recursos del Mar, Centro de Investigación y de Estudios Avanzados del IPN, Unidad Mérida, Yucatán 97310, Mexico; emanuel.hernandez@cinvestav.mx

[4] CAS Key Laboratory of Receptor Research, the National Center for Drug Screening, Shanghai Institute of Materia Medica, Chinese Academy of Sciences, Shanghai 201203, China, endlesslily@hotmail.com (Z.W.); xxie@simm.ac.cn (X.X.)

[5] Dipartimento di Biologia, Unità di Biochimica, University of Pisa, 56126 Pisa, Italy; mcappiello@biologia.unipi.it (M.C.); francesco.balestri@unipi.it (F.B.); umberto.mura@unipi.it (U.M.)

* Correspondence: gabriel_navarrete@uaem.mx; Tel.: +52-777-329-7089

† Taking in part of the Ph. D. thesis of Blanca Colín-Lozano.

REPRESENTATIVE SPECTRA

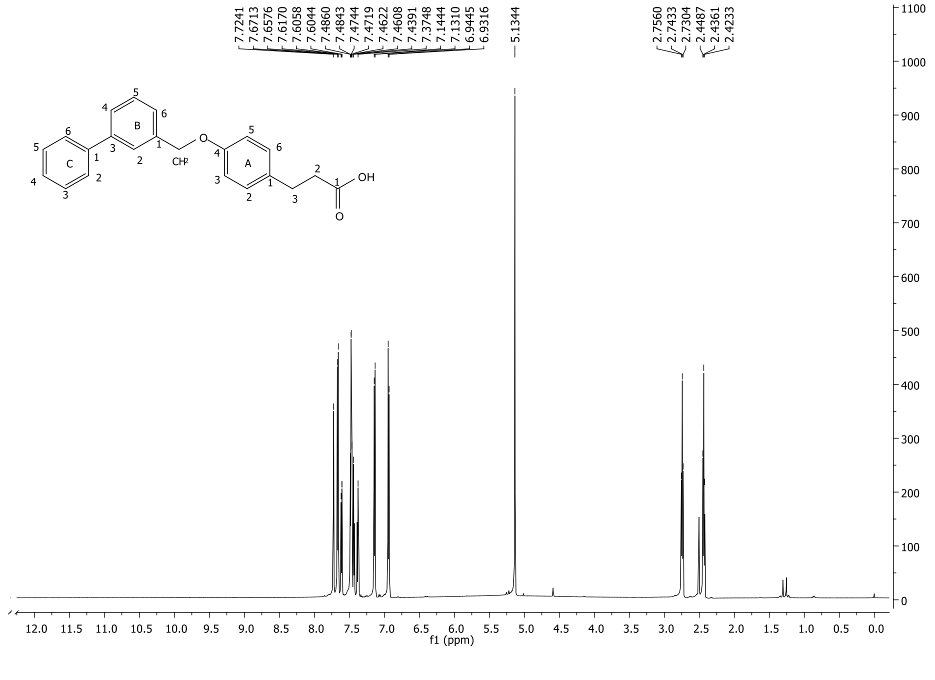

^{1}H-NMR of compound **1**

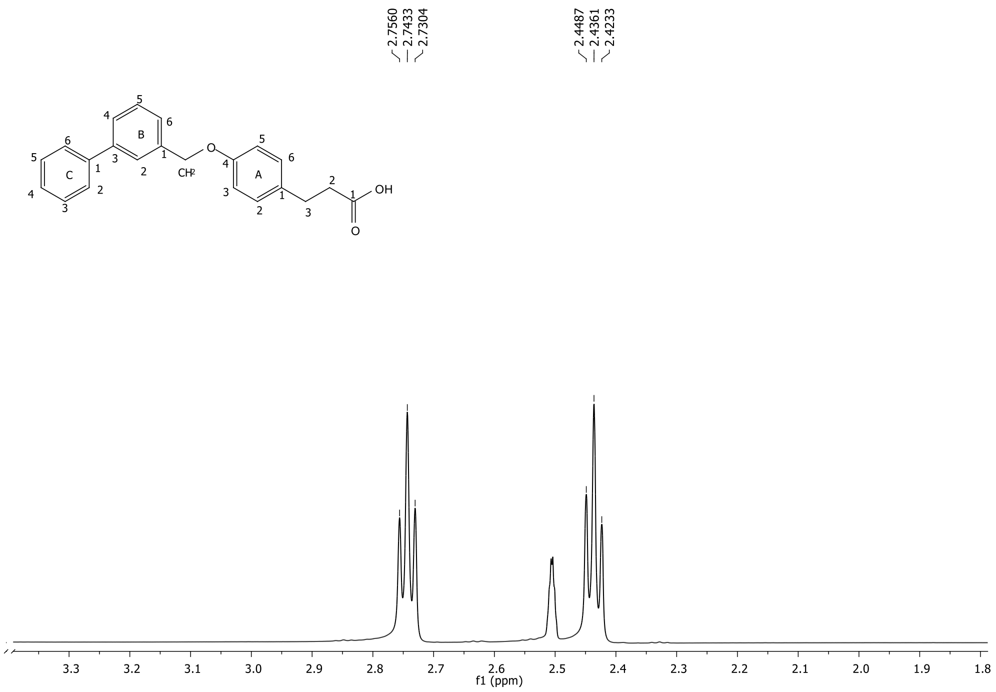

^{1}H-NMR of compound **1** (aliphatic zone expansion)

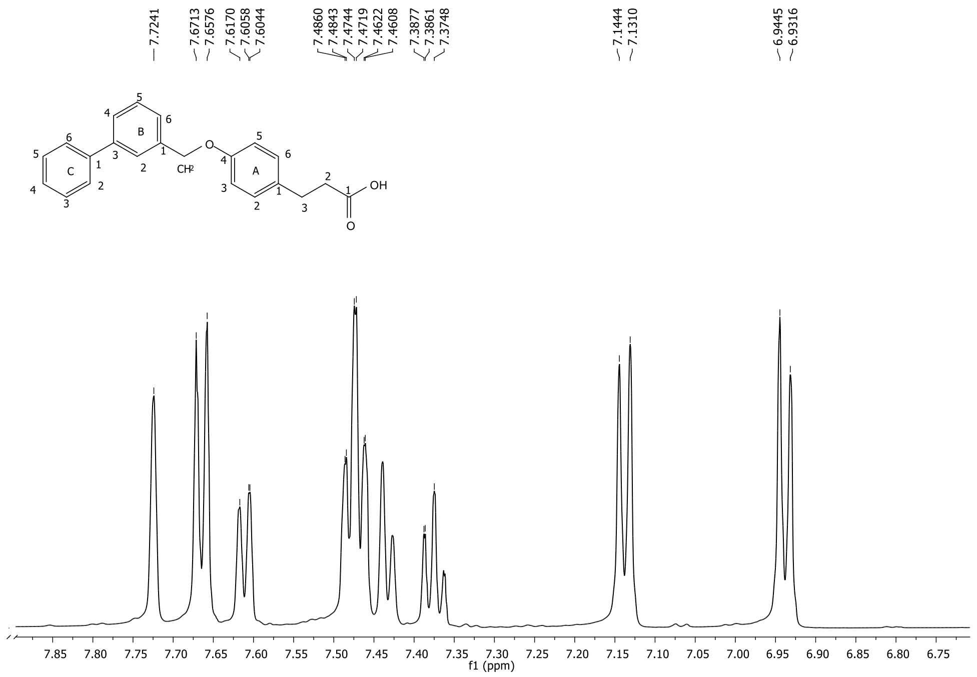

^{1}H-NMR of compound **1** (aromatic zone expansion)

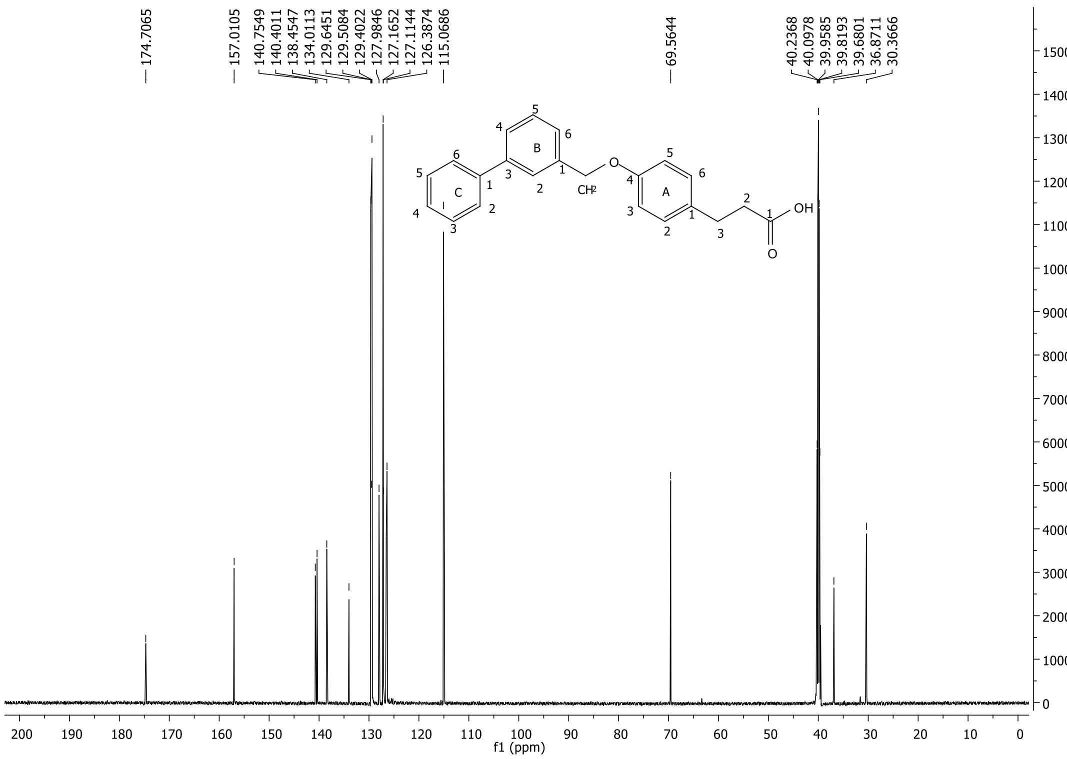

^{13}C-NMR of compound **1**

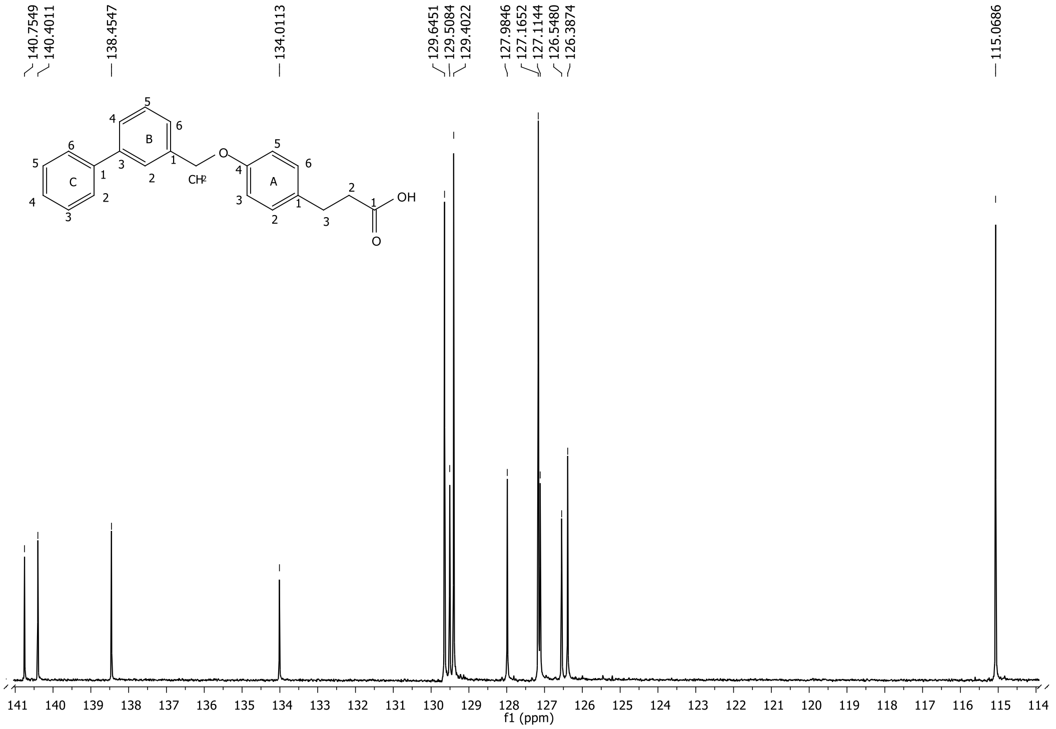

^{13}C-NMR of compound **1** (aromatic zone expansion)

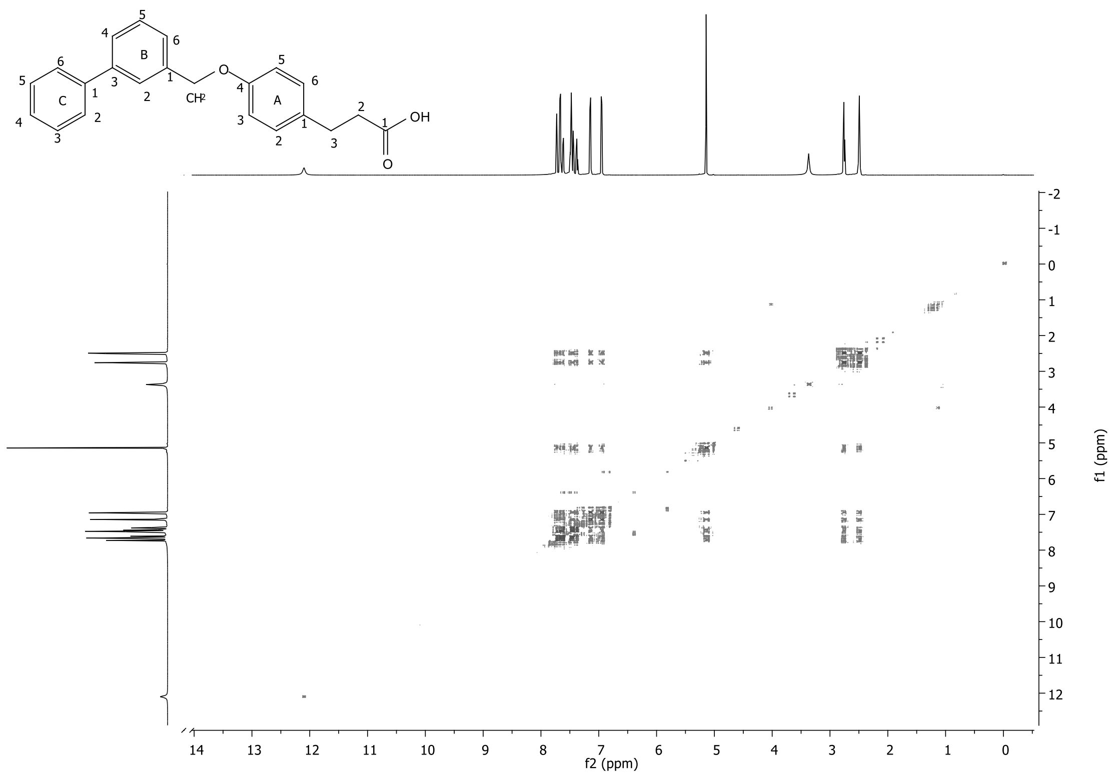

COSY of compound **1**

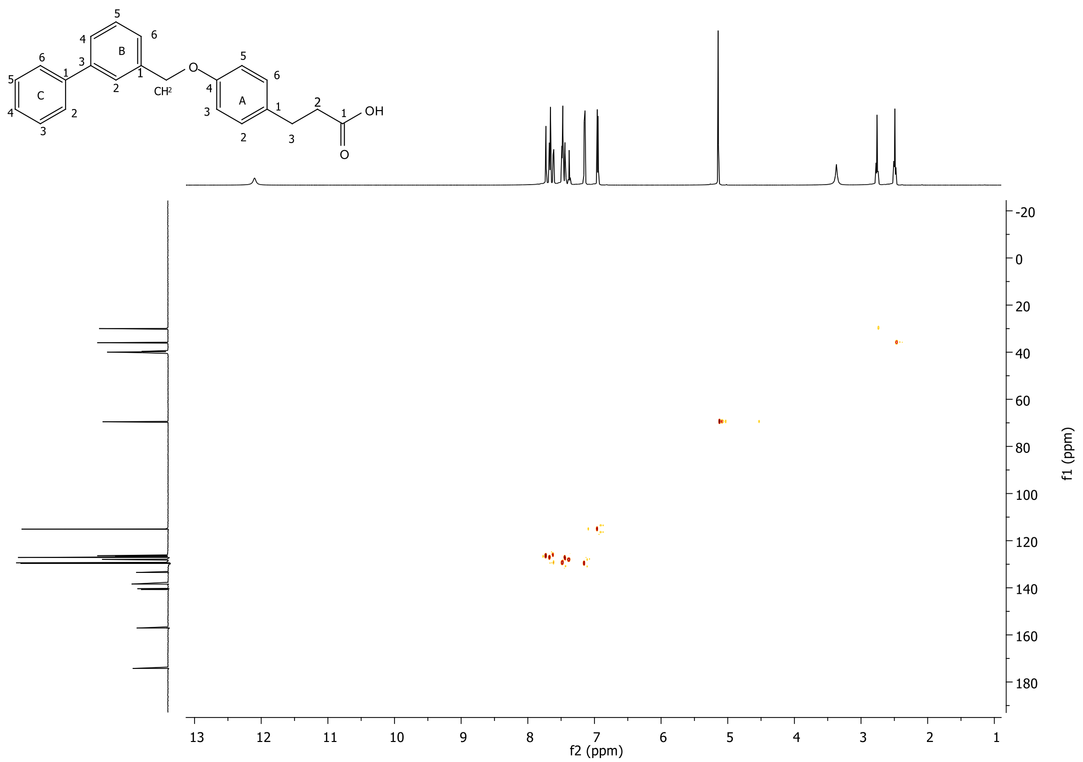

HSQC of compound **1**

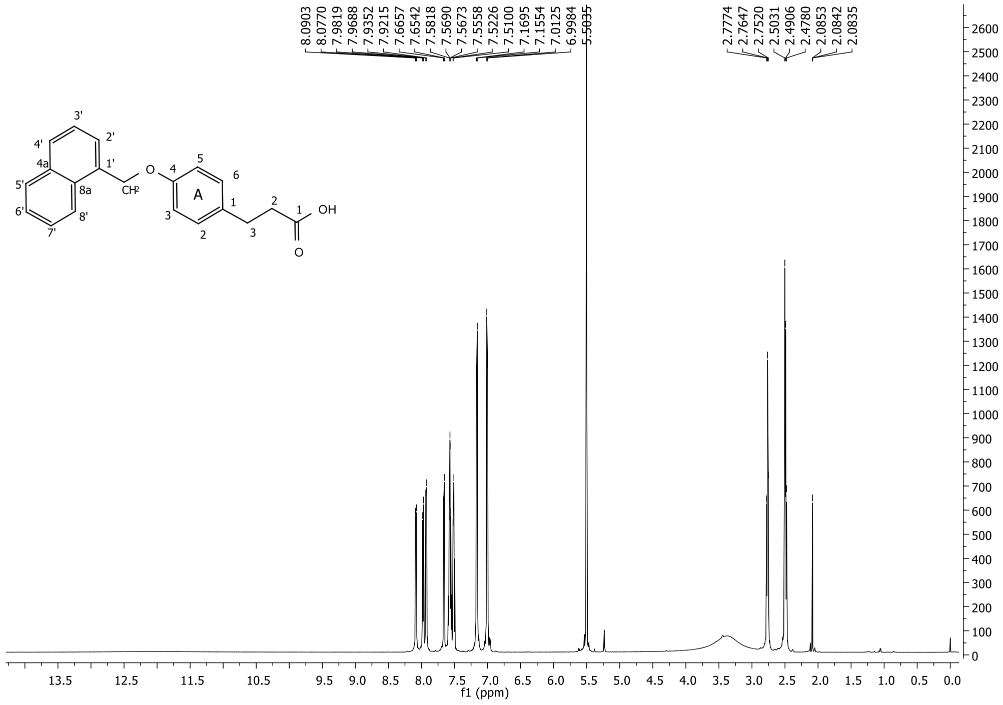

^{1}H-NMR of compound **2**

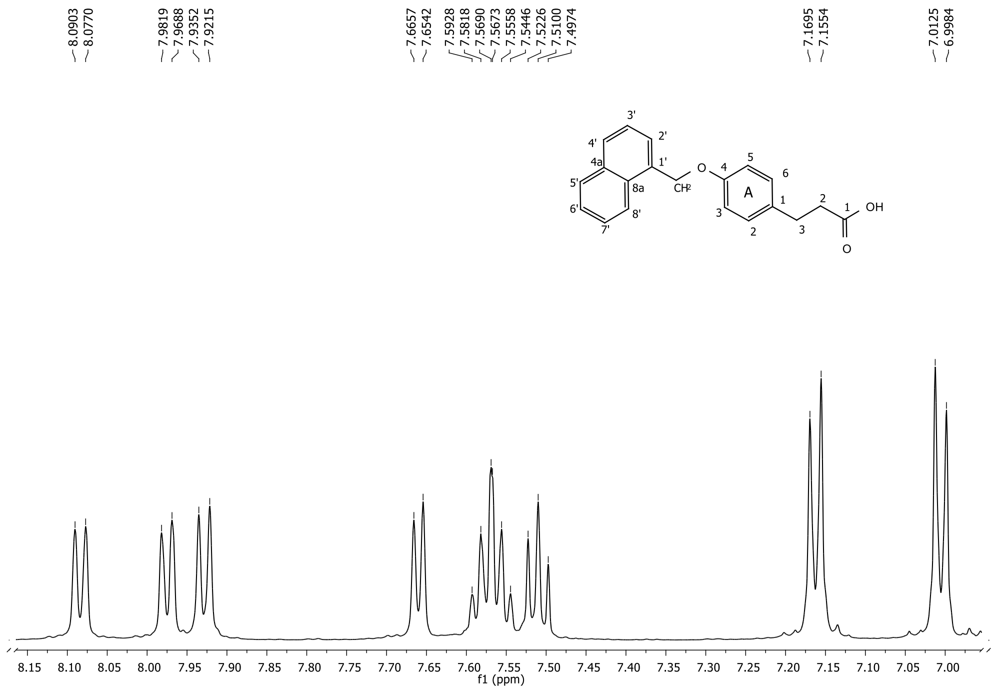

^{1}H-NMR of compound **2** (aromatic zone expansion)

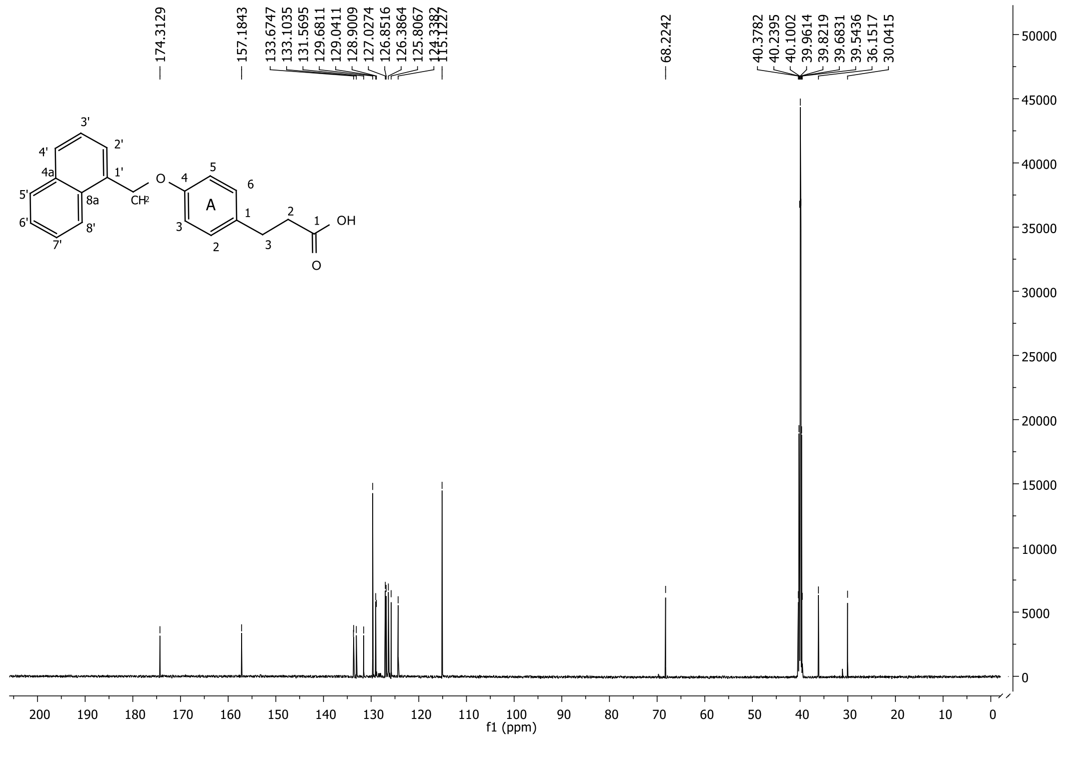

^{13}C-NMR of compound **2**

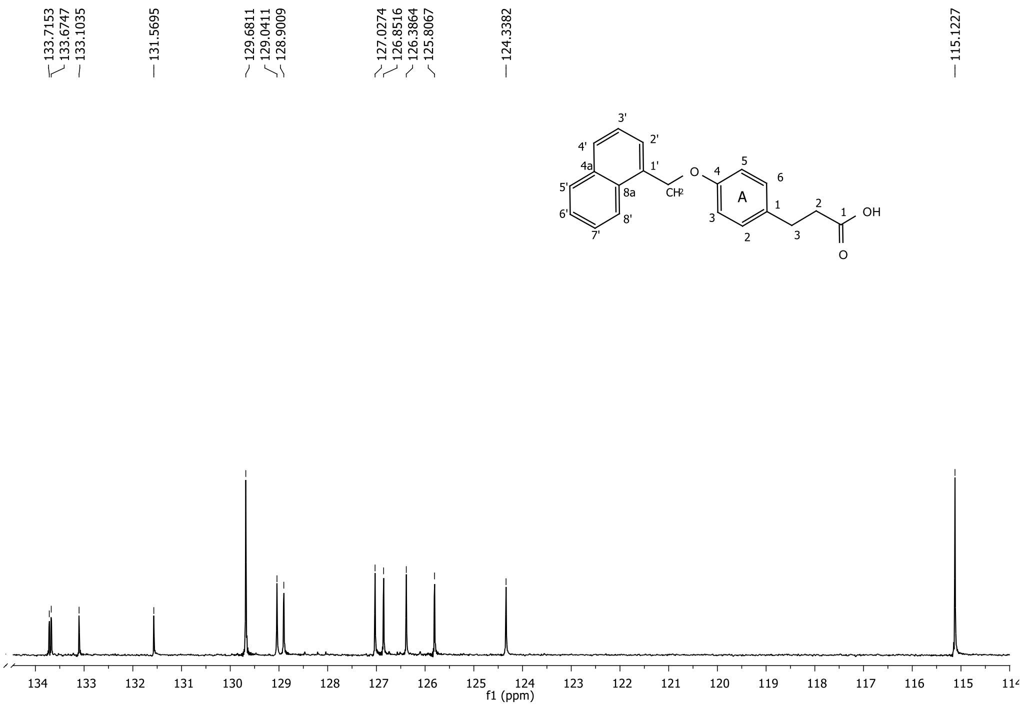

^{13}C-NMR of compound **2** (aromatic zone expansion)

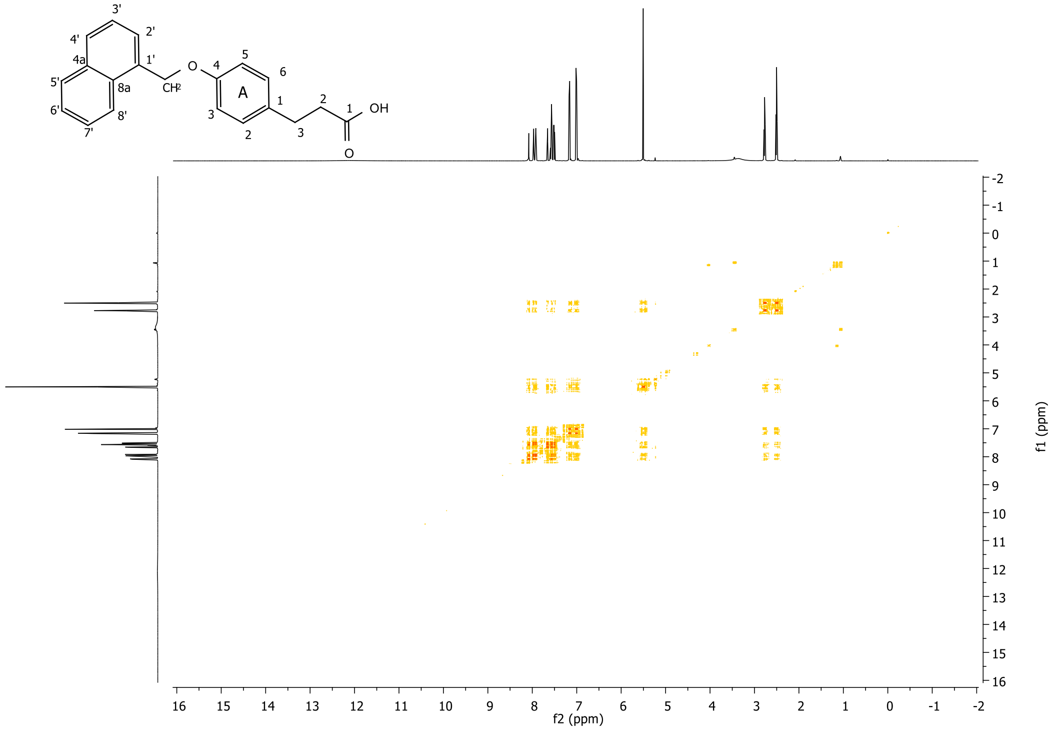

COSY of compound **2**

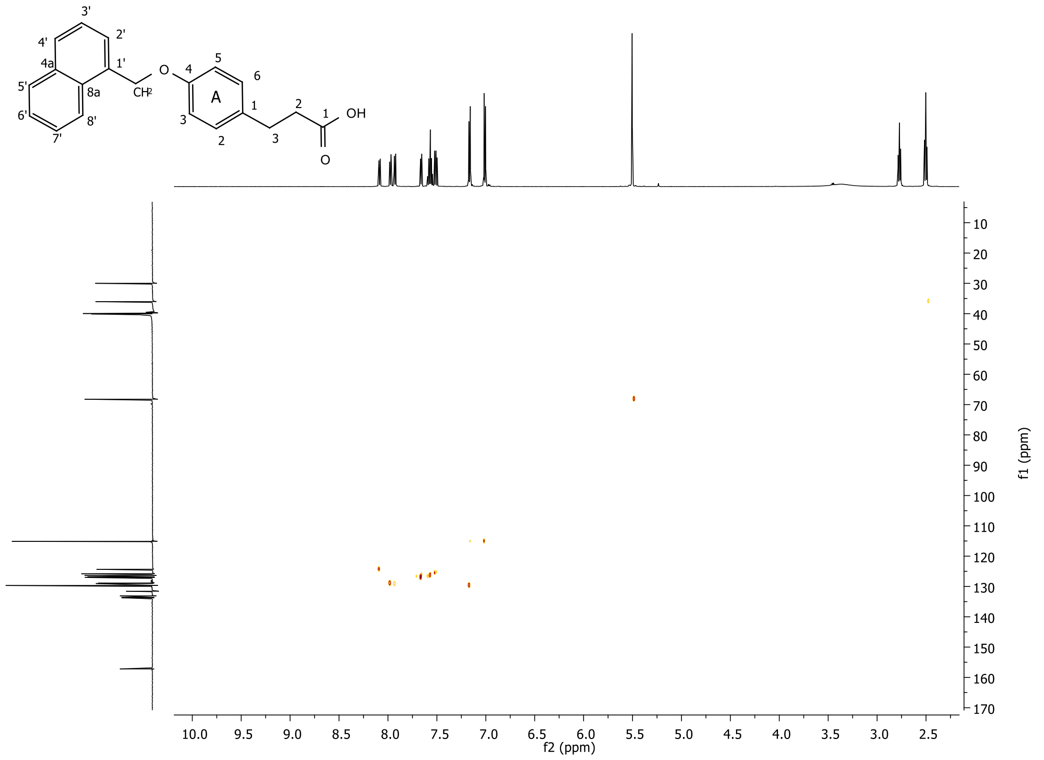

HSQC of compound **2**

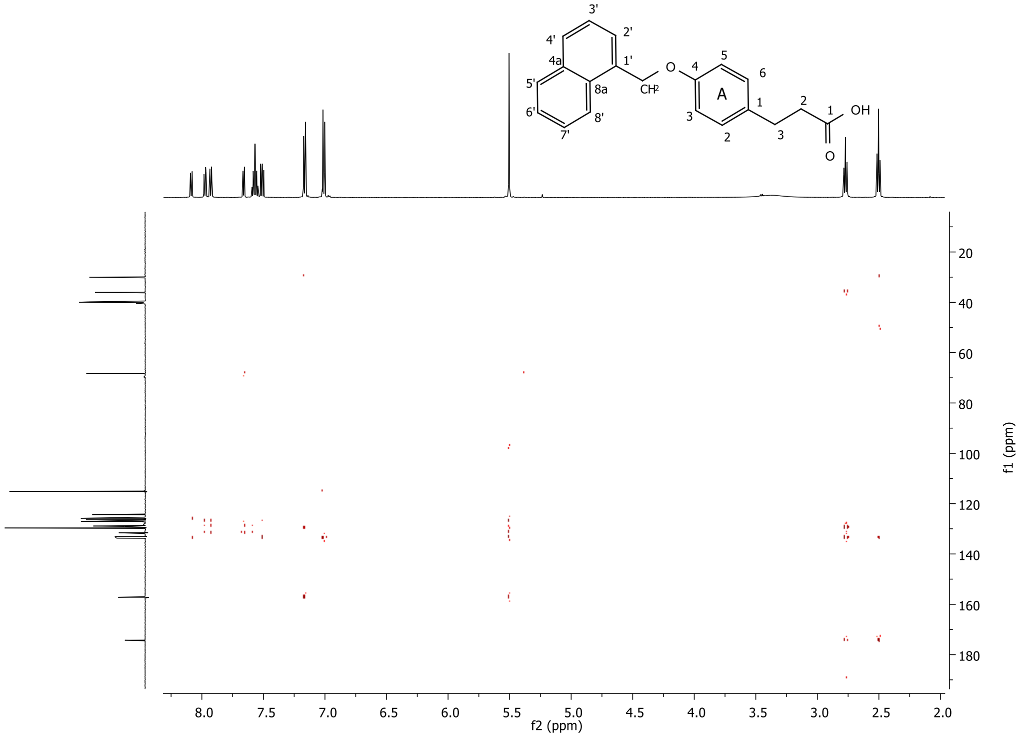

HMBC of compound **2**

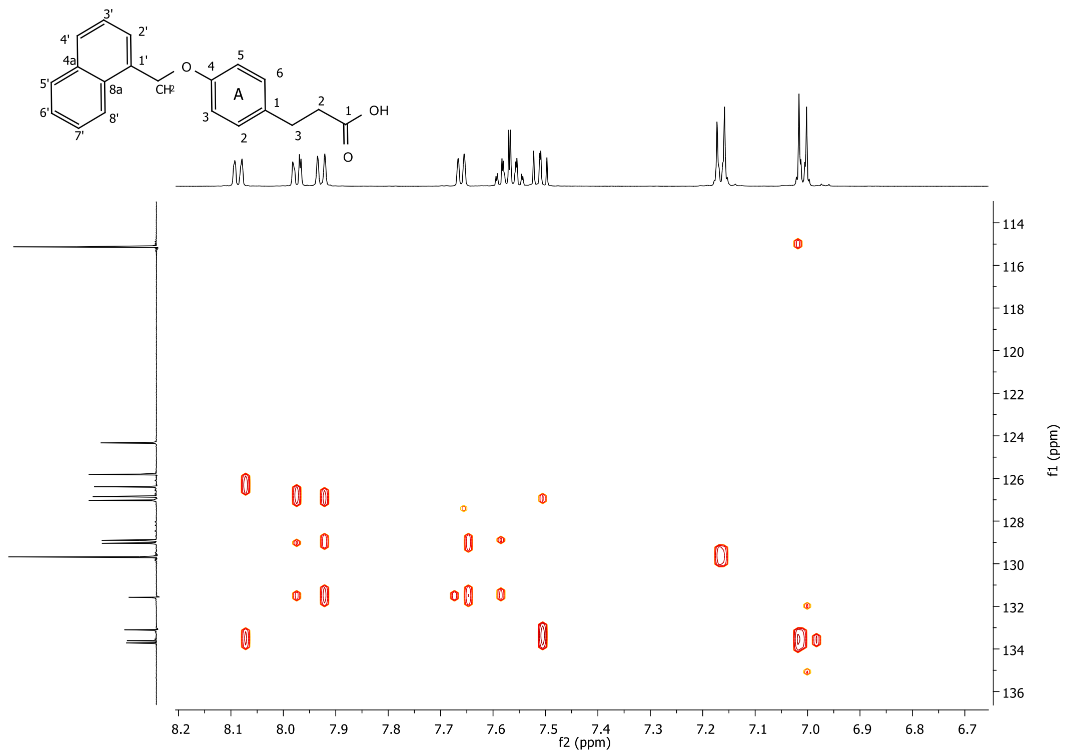

HMBC of compound **2** (expansion)

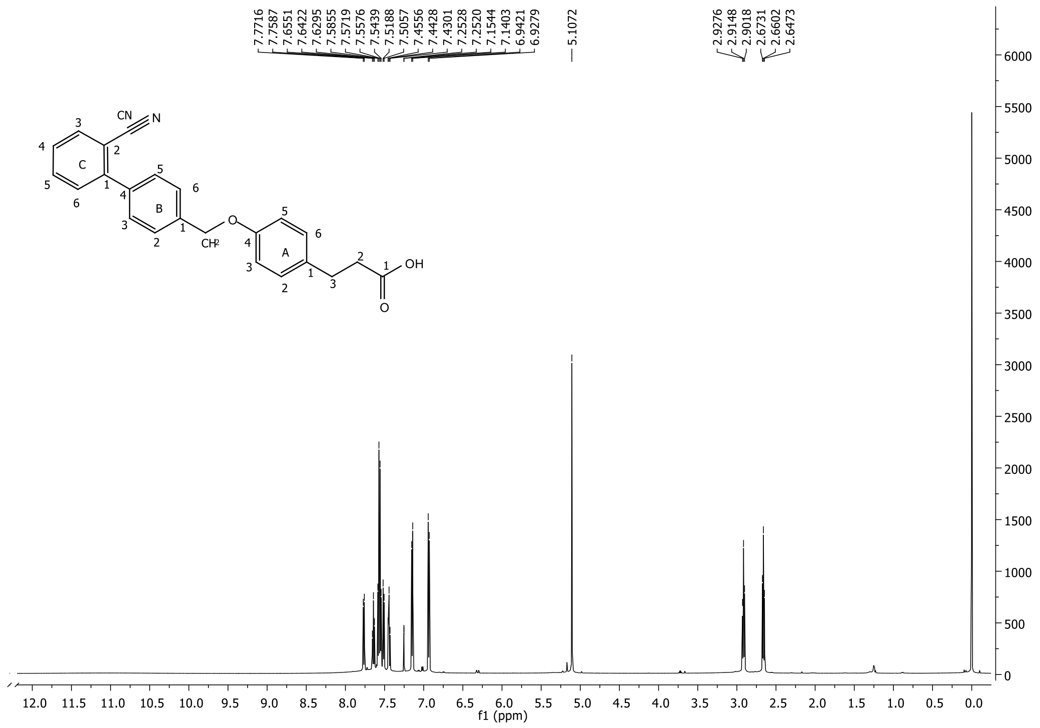

^{1}H-NMR of compound **3**

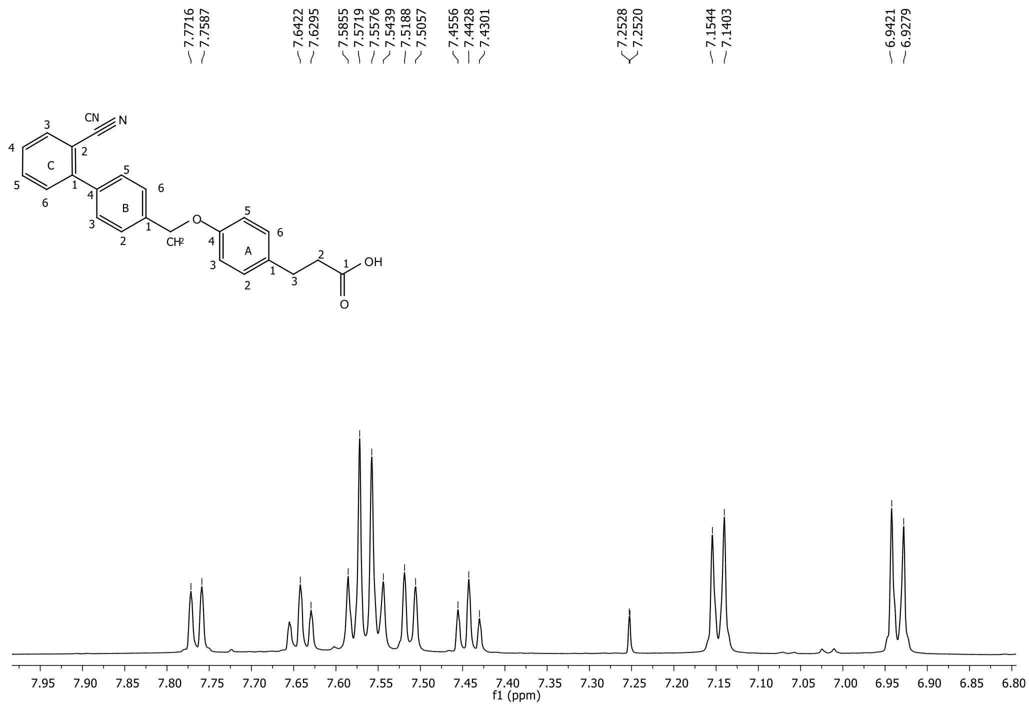

^{1}H-NMR of compound **3** (aromatic zone expansion)

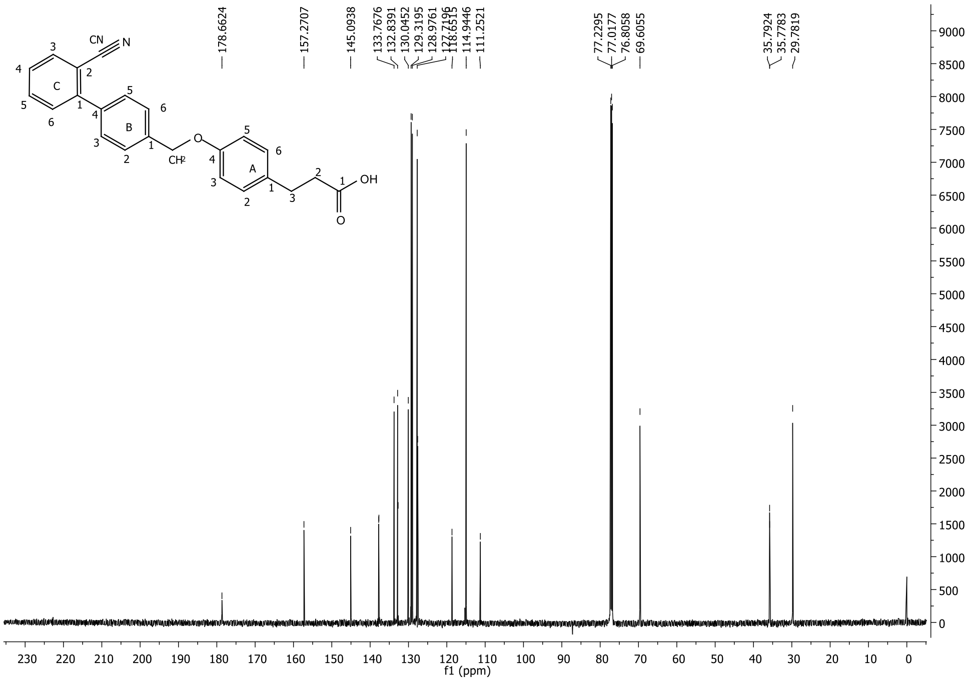

^{13}C-NMR of compound **3**

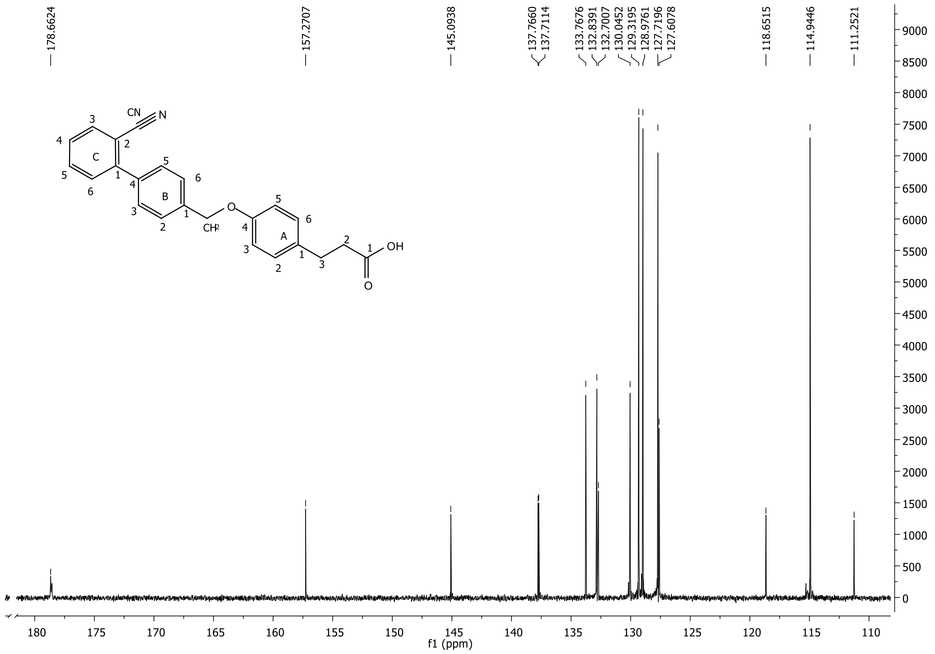

^{13}C-NMR of compound **3** (aromatic zone expansion)

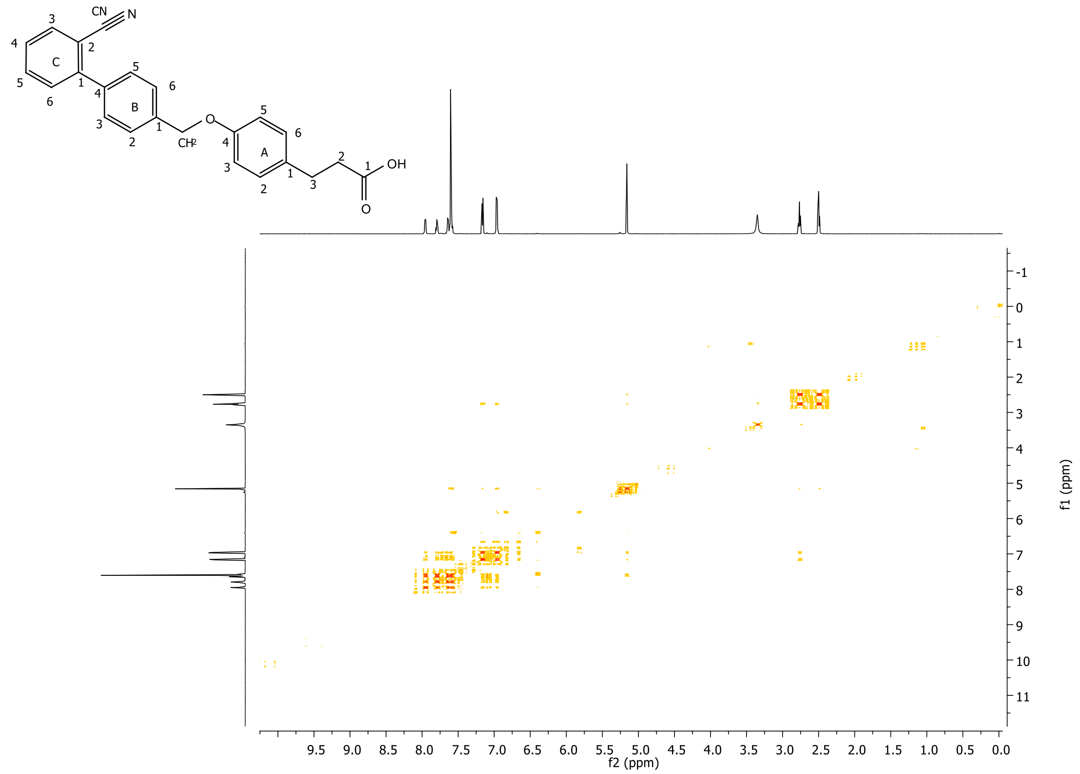

COSY of compound **3**

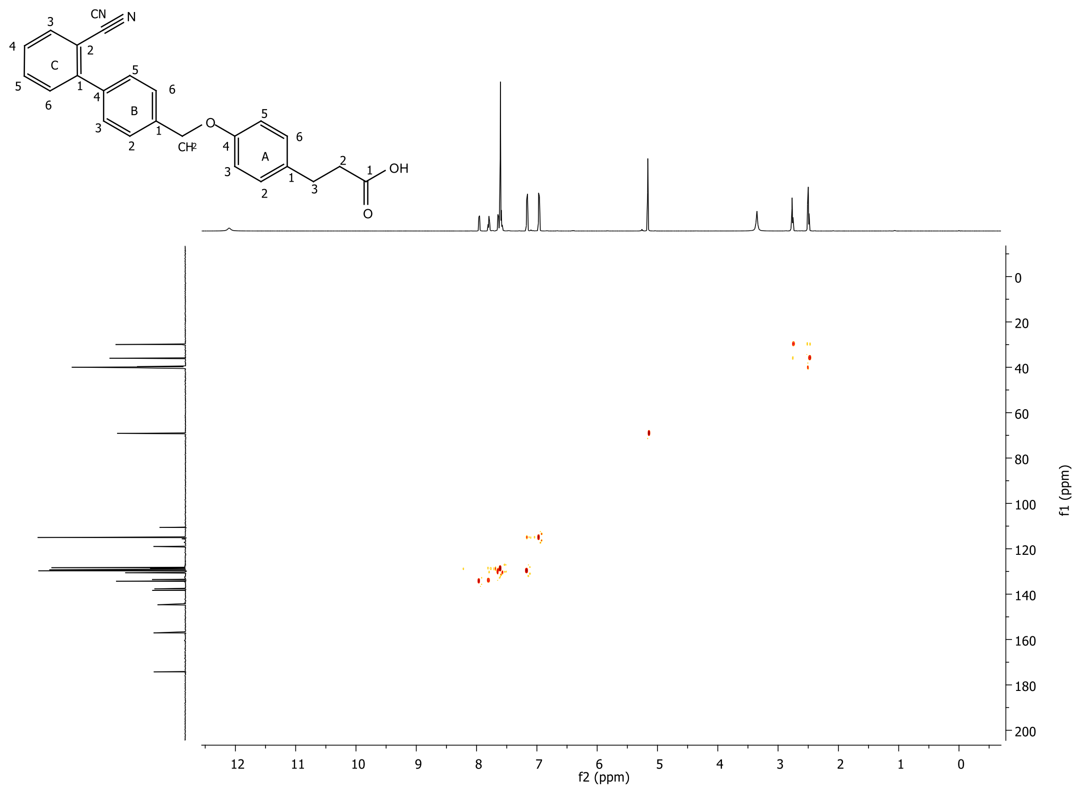

HSQC of compound **3**

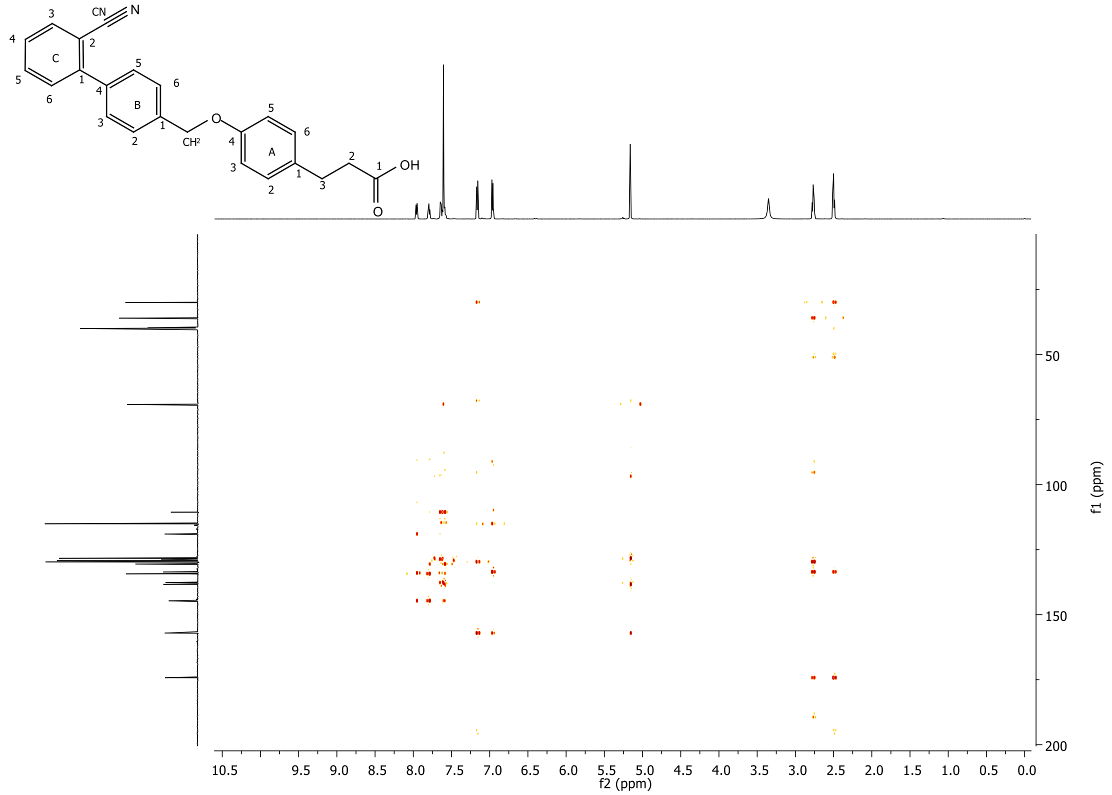

HMBC of compound **3**

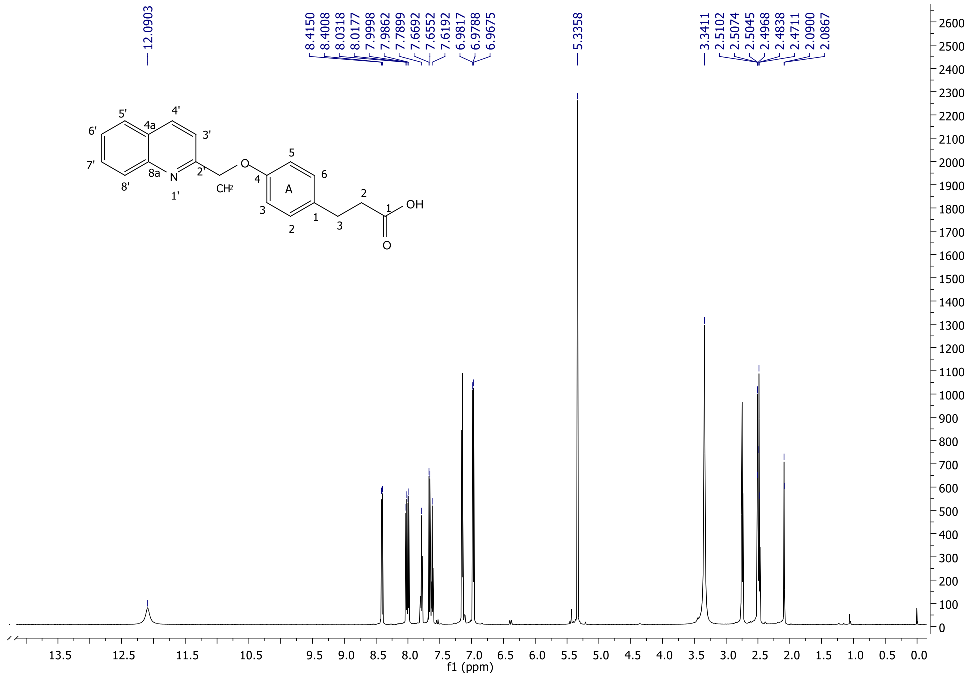

^{1}H-NMR of compound **4**

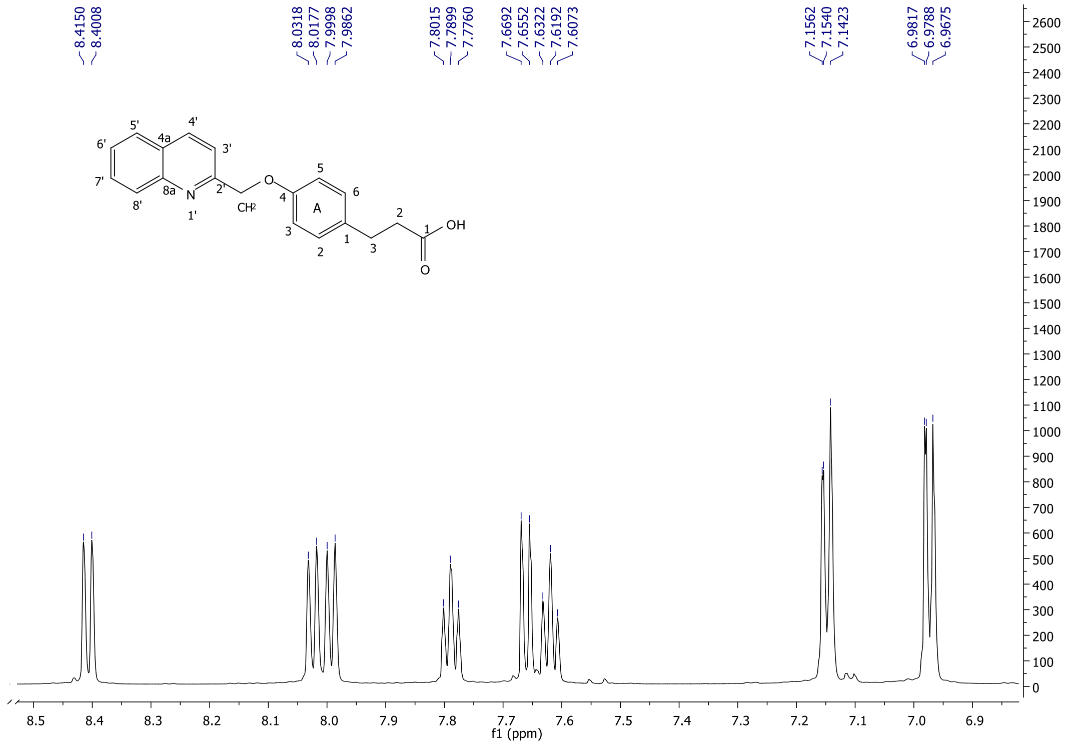

^{1}H-NMR of compound **4** (aromatic zone expansion)

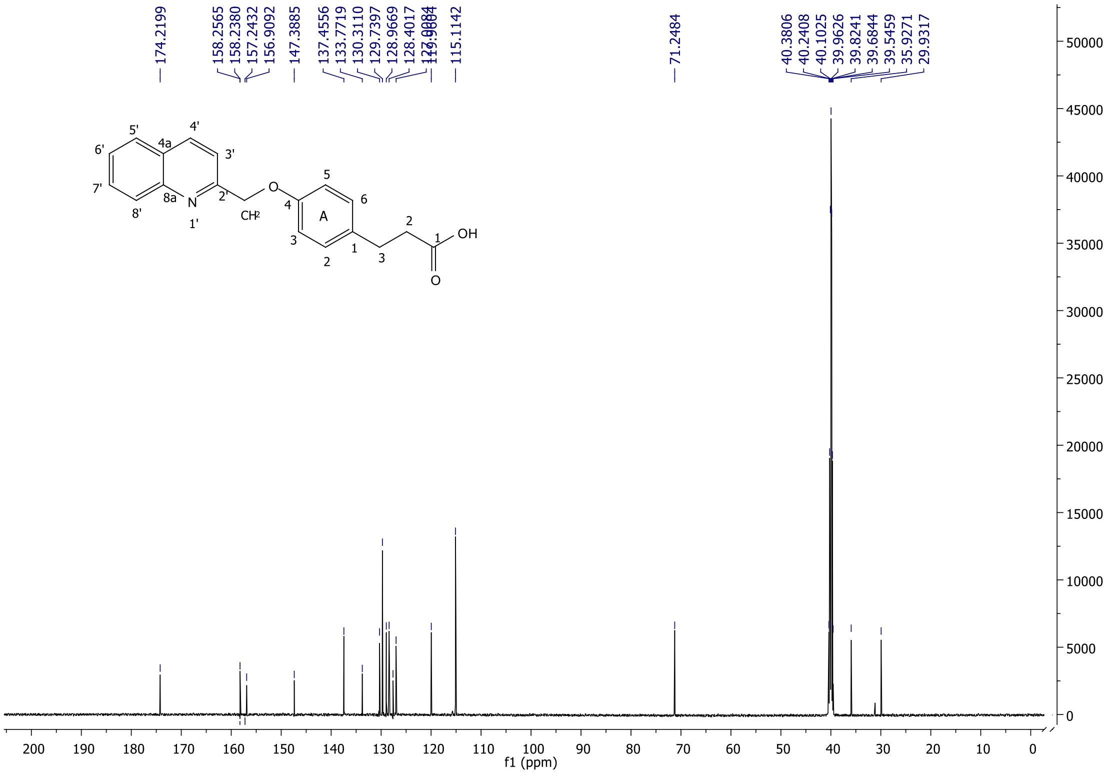

^{13}C-NMR of compound **4**

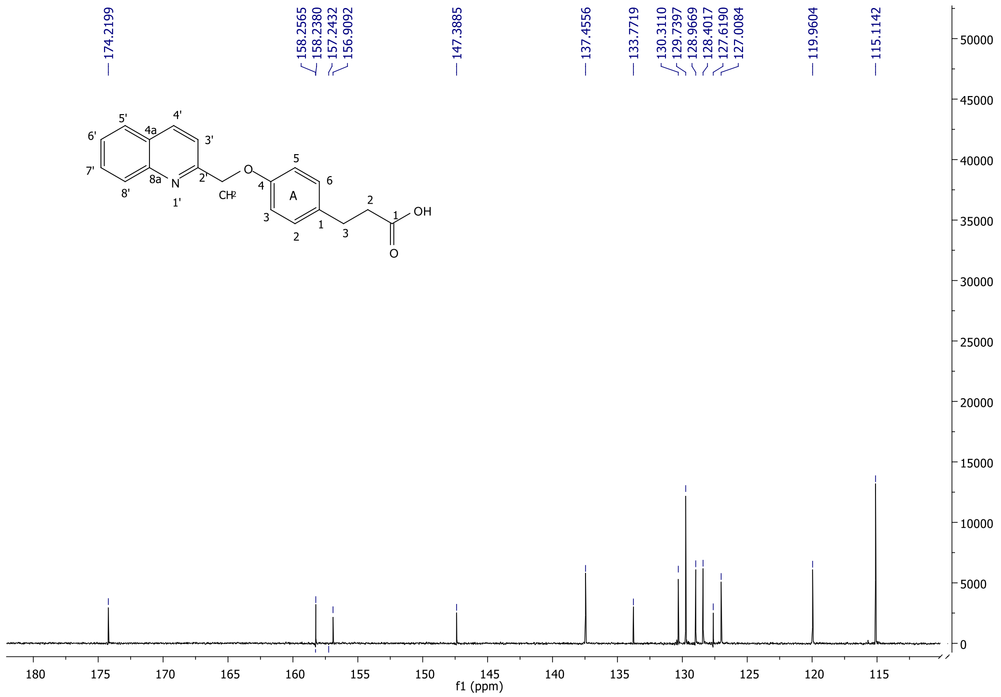

^{13}C-NMR of compound **4** (aromatic zone expansion)

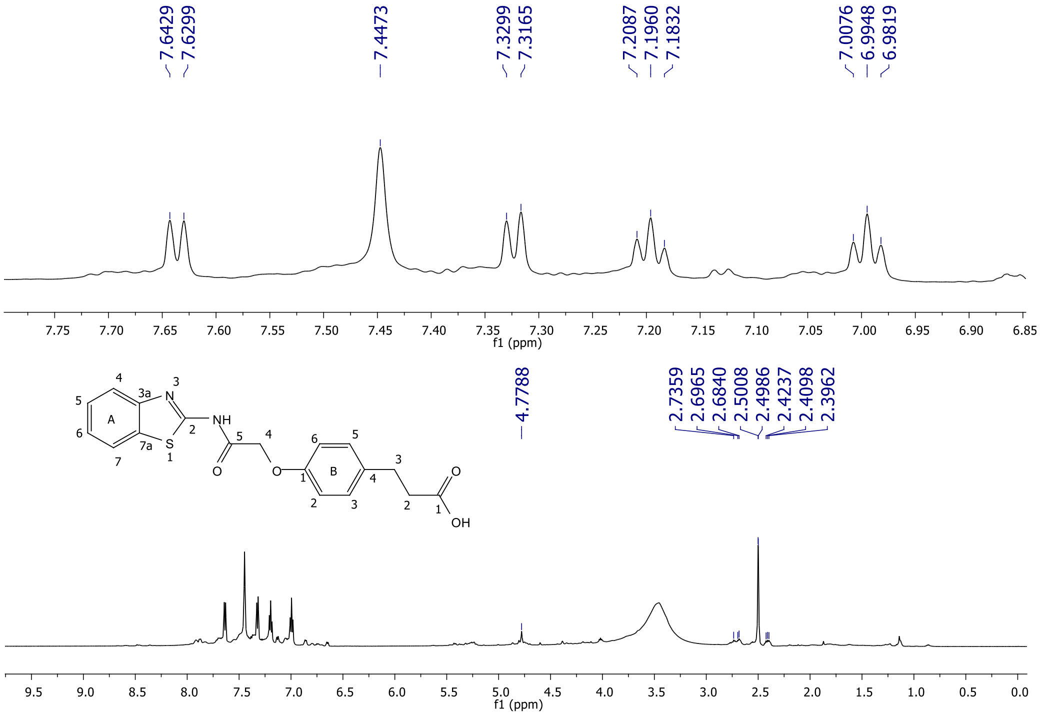

^{1}H-NMR of compound **5** (aromatic zone expansion)

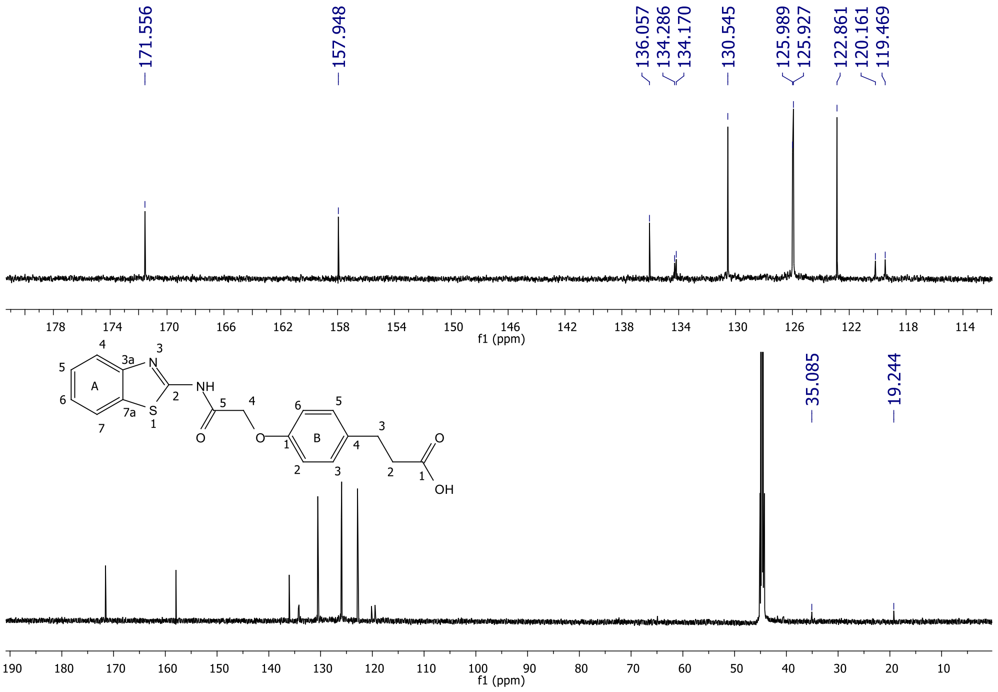

^{13}C-NMR compound **5** (aromatic zone expansion)
